# Genome-Scale Data Reveal Deep Lineage Divergence and a Complex Demographic History in the Texas Horned Lizard (*Phrynosoma cornutum*) throughout the Southwestern and Central United States

**DOI:** 10.1093/gbe/evab260

**Published:** 2021-11-26

**Authors:** Nicholas Finger, Keaka Farleigh, Jason T Bracken, Adam D Leaché, Olivier François, Ziheng Yang, Tomas Flouri, Tristan Charran, Tereza Jezkova, Dean A Williams, Christopher Blair

**Affiliations:** 1 Department of Biological Sciences, New York City College of Technology, City University of New York, Brooklyn, New York, USA; 2 Department of Biology, Miami University, Oxford, Ohio, USA; 3 Department of Biology, University of Washington, Seattle, Washington, USA; 4 Burke Museum of Natural History and Culture, University of Washington, Seattle, Washington, USA; 5 Faculty of Medicine, University Grenoble-Alpes, France; 6 Department of Genetics, Evolution and Environment, University College London, United Kingdom; 7 Department of Biology, Texas Christian University, Fort Worth, Texas, USA; 8 Biology PhD Program, CUNY Graduate Center, New York, New York, USA

**Keywords:** demography, introgression, lizards, phylogeography, speciation

## Abstract

The southwestern and central United States serve as an ideal region to test alternative hypotheses regarding biotic diversification. Genomic data can now be combined with sophisticated computational models to quantify the impacts of paleoclimate change, geographic features, and habitat heterogeneity on spatial patterns of genetic diversity. In this study, we combine thousands of genotyping-by-sequencing (GBS) loci with mtDNA sequences (ND1) from the Texas horned lizard (*Phrynosoma cornutum*) to quantify relative support for different catalysts of diversification. Phylogenetic and clustering analyses of the GBS data indicate support for at least three primary populations. The spatial distribution of populations appears concordant with habitat type, with desert populations in AZ and NM showing the largest genetic divergence from the remaining populations. The mtDNA data also support a divergent desert population, but other relationships differ and suggest mtDNA introgression. Genotype–environment association with bioclimatic variables supports divergence along precipitation gradients more than along temperature gradients. Demographic analyses support a complex history, with introgression and gene flow playing an important role during diversification. Bayesian multispecies coalescent analyses with introgression (MSci) analyses also suggest that gene flow occurred between populations. Paleo-species distribution models support two southern refugia that geographically correspond to contemporary lineages. We find that divergence times are underestimated and population sizes are overestimated when introgression occurred and is ignored in coalescent analyses, and furthermore, inference of ancient introgression events and demographic history is sensitive to inclusion of a single recently admixed sample. Our analyses cannot refute the riverine barrier or glacial refugia hypotheses. Results also suggest that populations are continuing to diverge along habitat gradients. Finally, the strong evidence of admixture, gene flow, and mtDNA introgression among populations suggests that *P. cornutum* should be considered a single widespread species under the General Lineage Species Concept.

Significance

Many studies have documented cryptic diversity in diverse taxa inhabiting the arid regions of western North America, with divergence correlated with both Neogene vicariance and Pleistocene climate change. However, relatively few studies adopt a genomics approach and most implicitly assume that gene flow ceases once divergence begins. Using the Texas horned lizard (*Phrynosoma cornutum*) as a model, our results suggest a complex demographic history that includes episodes of gene flow. Results also suggest that divergence is continuing along environmental axes and that adequate model choice is imperative for demographic hypothesis testing. This study can serve as a model for how genomic data and new analytical tools can be used to test traditional evolutionary hypotheses throughout geologically and climatically diverse regions.

## Introduction

Allopatric divergence has long been considered the most likely cause of speciation, and geographic barriers the primary hindrance to gene flow (Coyne and Orr 2004). However, the origins of a particular diversification event can be both controversial and unclear, resulting in the various forces behind diversification becoming a current topic for discussion (Butlin et al. 2008; Fitzpatrick et al. 2009; Pyron and Burbrink 2010; Nosil and Feder 2012). Not only may the forces acting on species be disparate, but the diversification process can be episodic with periods of isolation interspersed with periods of gene flow leading to a history of reticulation (Blair and Ané 2020). As the climate changes, a population may fracture by seeking shrinking patches of ideal habitat, expand into newly habitable regions, or adapt, the latter of which can lead to niche divergence and ecological segregation (Wiens and Graham 2005; Jezkova et al. 2016; Castro-Insua et al. 2018). As a species expands or contracts its range, it may encounter hard barriers to gene flow such as rivers, which have been shown to result in genetic divergence in multiple taxa (Pastorini et al. 2003; Nazareno et al. 2019). Populations and species likely to encounter disruptive barriers throughout their history tend to occupy a wide geographic range of varied habitat, yet possess low dispersal capabilities (Schield et al. 2018). Ectothermic species such as reptiles that exhibit these traits are also further influenced by climate differences (Huey and Kingsolver 1993; Wogan and Richmond 2015). Ultimately, understanding the evolutionary history of a species involves evaluating the geographic, genetic, and climatic factors affecting divergence throughout its history (Fitzpatrick et al. 2009).

The Texas horned lizard (*P.cornutum*) is spread across a diverse collection of ecological habitats making it an interesting candidate to examine adaptation and phylogeographic history. Although its range does consist of many smaller environmental niches (Price 1990), there exists a primary habitat divide that bisects the species’ distribution providing an apparently stark environmental contrast through which to view its effects on the species. The southwestern range inhabits the Chihuahuan desert of AZ and NM, whereas the northeastern range covers the Great Plains east of the Rocky Mountains throughout TX, OK, and KS extending the furthest east of any horned lizard (Sherbrooke 2003). As expansive as the range is, *P. cornutum* lives a sedentary life, maintaining fidelity to a home range with daily movement <250 m and limited long distance dispersal capabilities (Fair and Henke 1999). The combined factors of the species’ large geographic distribution, low dispersal ability, and varied ecological niche (with respect to various environmental variables such as temperature and precipitation) across the range may increase the likelihood of regional adaptation (Lenormand 2002; Newman and Austin 2015). Of particular note is the broad range of annual precipitation values, from approximately 10 in per year in the western deserts to approximately 50 in per year in the Great Plains (Pittman et al. 2007). *Phrynosoma cornutum* has also developed mechanisms for water harvesting involving both behavioral and morphological adaptations (Sherbrooke 1990). The lizard will adopt a rain-harvesting stance, spreading the dorsal surface so as to maximize retention of raindrops which are then carried through interscalar channels to the mouth (Sherbrooke 1990). These behavioral and morphological adaptations are shared with other *Phrynosoma* (*Phrynosomamodestum* and *Phrynosomaplatyrhinos*) inhabiting similar arid ecological niches (Sherbrooke 1990; Sherbrook 2003), and suggest that there may be clines in allele frequencies that are partially tied to temperature and/or precipitation.

With the uniqueness of these adaptations, along with their status in historical accounts and importance in use as symbols and mascots, *Phrynosoma* spp. have been the subject of interest in many evolutionary studies (Leaché and McGuire 2006; Leaché and Linkem 2015; Williams et al. 2019). The crown group of *Phrynosoma* diverged roughly 25 Ma and the genus now contains 17 species after the addition of three new species over the past decade. Recent studies focusing on the genetic structure and lineage divergence within the various species (Mulcahy et al. 2006; Bryson et al. 2012; Montanucci 2015; Jezkova et al. 2016; Blair and Bryson 2017) yielded the discovery of these three new additions, *Phrynosomacerroense*, *Phrynosomablainvilli*, *andPhrynosomasherbrookei*, to the taxonomy (Leaché et al. 2009; de Oca et al. 2014). Previously, relationships both between and within species have been difficult to untangle due to hybridization, introgression, and incomplete lineage sorting (ILS) resulting in disagreement between concatenation versus coalescent-based methods, as well as discordance between trees inferred using mitochondrial DNA (mtDNA) and nuclear DNA (nDNA) (Leaché and McGuire 2006; de Oca et al. 2014). With the advent of reduced representation sequencing providing a random and more diverse view of the genome (Andrews et al. 2016), we are able to overcome these previous challenges in discerning phylogenetic and phylogeographic relationships caused by mtDNA introgression and gene tree/species tree discordance (Leaché and Linkem 2015; Leaché et al. 2015). Given the comparatively large geographic range of *P. cornutum*, and the lack of genomic assessment across diverse habitats, the possibility of cryptic diversity is high.

A previous study of this species found strong divergence between the western desert and eastern plains populations using mtDNA data (Williams et al. 2019). It was hypothesized that the presence of an extensive late Pliocene pluvial lake, Lake Cabeza de Vaca, was the barrier that originally separated these two clades. Both clades gave a signal of population expansion in the Pleistocene. Nuclear microsatellite loci also revealed strong divergence between the western and eastern mitochondrial clades and found that the eastern plains were further subdivided into the South-Central Semi-Arid Prairies to the north of the Balcones Escarpment and the Southern Texas Plains south of the Escarpment (Williams et al. 2019). Although these results further advance our understanding of evolutionary pattern and process throughout the central-southern United States, a genomic approach that takes advantage of sophisticated new analytical tools would provide additional power to disentangle competing hypotheses regarding historical and contemporary divergence.

In this study, we expand on previous results by including samples from more northern areas of the species range (KS and OK) and by examining the phylogeographic and demographic history of *P. cornutum* using both mtDNA sequences and thousands of nuclear SNPs from a modified genotyping-by-sequencing (GBS) approach. We first use concatenated and coalescent-based phylogenetic analyses, species delimitation analyses, and clustering to test the hypothesis that the genomic and mtDNA data support the presence of cryptic diversity, which has been demonstrated in other species of *Phrynosoma* with large geographic distributions. Second, we use genotype–environment association (GEA) analyses to test the hypothesis that a proportion of SNPs are statistically correlated with bioclimatic variables and that the environmental gradient between the plains and desert habitat may be driving adaptation and furthering genetic divergence (McDonald 1983; Wiens et al. 2013). We then adopt an explicit hypothesis testing framework to elucidate demographic history, testing three hypotheses of divergence likely important to the species. Specifically, we use our models to assess the relative importance of the Rio Grande as a hard allopatric barrier to gene flow between divergent lineages (Lanna et al. 2020), as compared with soft allopatric divergence due to cyclical paleoclimate change or ecological gradients. Both present day and historical species distribution models (SDMs) are used to further test the hypothesis that divergence was driven by Pleistocene climate fluctuations (Hewitt 1996, 2000) as has been observed for other inhabitants in the region (Schield et al. 2015; Jezkova et al. 2016). Finally, we test the hypothesis that explicitly accommodating gene flow in Bayesian multispecies coalescent analyses (MSci; Flouri et al. 2020), leads to alternative estimates of demographic history (i.e., divergence times and effective population sizes).

## Results

### Data Set Characteristics

We obtained approximately 225 megabases of normalized GBS (nGBS) data from 75 *P. cornutum* samples and a single *Phrynosomasolare* outgroup. After processing the data in ipyrad (Eaton and Overcast 2020), most individuals had approximately 30,000 loci (4,757–42,652; supplementary table S1, Supplementary Material online). The full-concatenated matrix consisted of 7,906,017 bp and 57,459 loci. The final mtDNA alignment consisted of 1,330 bp, 119 variable (but parsimony uninformative) sites, and 101 parsimony informative sites across 74 sequences including a single *P. solare* sequence used as the outgroup. Excluding the outgroup resulted in 27 variable (parsimony uninformative) sites and 100 parsimony informative characters.

### Phylogenetic Analysis

**Fig. 1.**
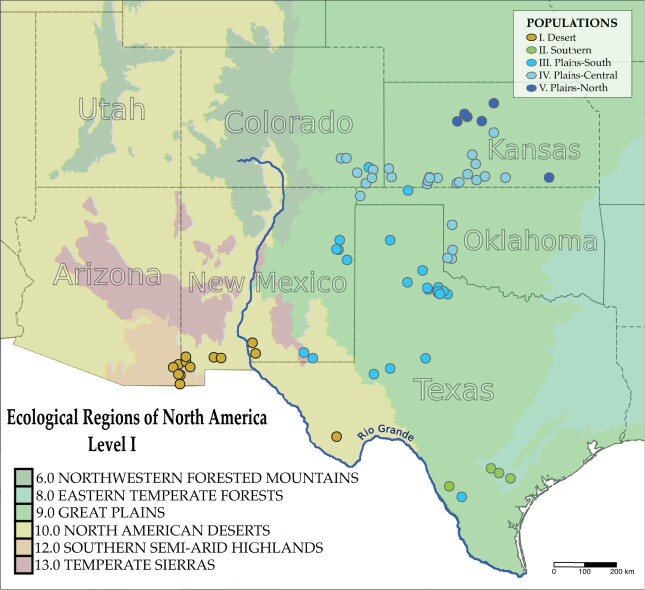
Sample locations for all *Phrynosoma cornutum* used in this study within the EPA level I ecoregions. Population assignments are based on genotypes from the nGBS data set using the program sNMF.

**Fig. 2.**
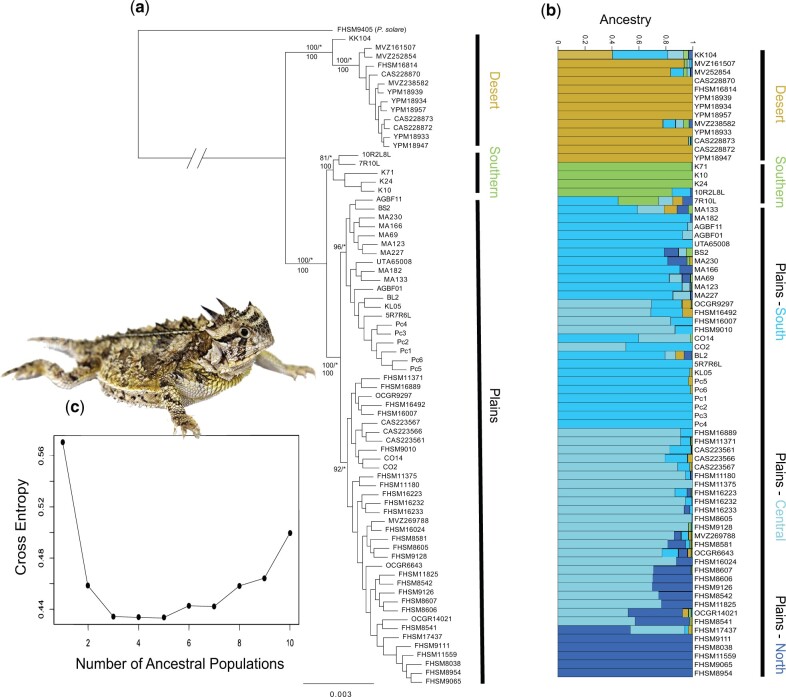
(*a*) Maximum likelihood genealogy inferred using RAxML-ng on a concatenated nGBS matrix of 7,906,017 bp. Values at nodes (on top) represent ML bootstrap proportions/Bayesian posterior probabilities from ExaBayes (*=1.0). Values at nodes (below) represent bootstrap support (100 replicates) from SVDquartets analyses on a matrix of 54,634 SNPs. The branch leading to the outgroup was pruned for clarity. (*b*) Population structure inferred using sNMF. (*c*) The cross-entropy criterion supported five ancestral populations (*K* = 5).

We used multiple phylogenetic analyses to test for the presence of cryptic lineages and elucidate the relationships among them. Concatenated maximum likelihood (ML) analysis in RAxML-ng (Kozlov et al. 2019) yielded a topology consisting of three primary lineages (figs. 1 and 2). These lineages included a Desert clade (DST) consisting of samples from the AZ and NM portions of the Chihuahuan Desert (N. American Eco Region 10: North American desert), a Southern clade (STH) containing samples from the southern Texas plains (N. American Eco Region 9: Great Plains) and a Plains clade (PLN) of samples from Western NV, Northern TX, CO, KS, and OK (N. American Eco Region 9: Great Plains; fig. 1). The Desert lineage was supported by a bootstrap value of 100%, the Southern Lineage had a bootstrap value of 81% and the Plains lineage was also supported by 100% bootstrap value. The average relative Robinson–Foulds distance in this tree set was 0.079466 and the number of unique topologies in the tree set was 10. In all cases, the three primary clades were recovered. Bayesian analysis in ExaBayes (Aberer et al. 2014) resulted in a nearly identical topology to the ML tree with 100% posterior probability for the three distinct lineages (fig. 2). ESS values for all parameters indicated that the chain was run for an adequate duration (ESS>200 for all parameters). Both the ML and Bayesian analyses provided some additional support for two lineages within the Plains clade. The bootstrap consensus tree from SVDquartets (Chifman and Kubatko 2014) yielded a topology consistent with the ML and Bayesian trees (fig. 2). Bootstrap support for each clade was 100%. However, this topology did not support two distinct Plains lineages (supplementary fig. S1, Supplementary Material online).

Bayesian analysis of the mtDNA data in BEAST (Bouckaert et al. 2019) yielded high ESS values for all parameters (>200). The coefficient of variation parameter under a relaxed clock model (which measures the extent of clock violation) had substantial posterior density near zero, indicating that a strict clock model was appropriate. The maximum clade credibility tree showed a different tree topology compared with the three GBS-based trees discussed above. The Desert clade was still present and strongly supported (minus sample KK104), but the remaining topology did not support a distinctive Southern or Plains population. Instead, individuals from the Southern and Plains populations were interspersed throughout two lineages that diverged approximate 1 Ma (assuming a substitution rate of 0.00805 substitutions per site per million years; Macey et al. 1999). The mtDNA genealogy supported an initial divergence time of approximately 5 Ma for *P. cornutum* (supplementary fig. S2, Supplementary Material online).

### Population Structure and GEA Analyses

**Fig. 3.**
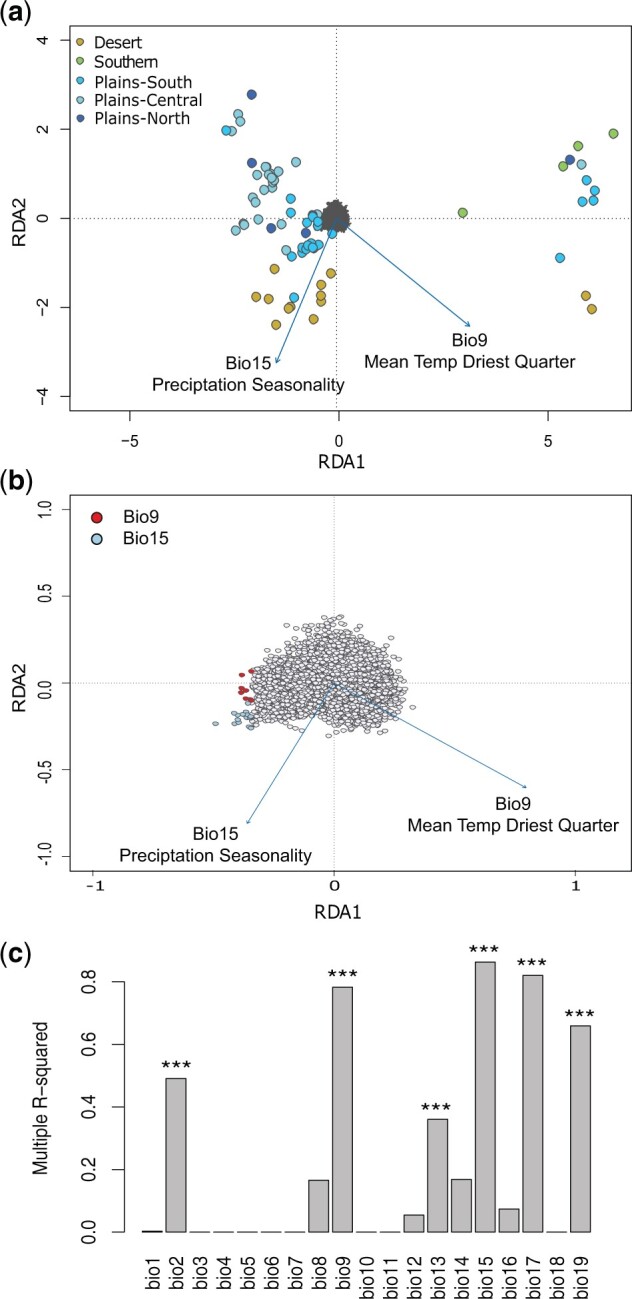
Results from the GEA analyses. Plots from the RDAs for the first two constrained ordination axes. (*a*) Relationship between individuals from the sNMF population assignments (color-coded circles) and the tested environmental variables (arrows). (*b*) Outlier loci (color-coded to environmental variable) and directionality of the relationship between the climate variables (arrows). (*c*) Importance of environmental variables in LFMM analysis as indicated by *P* values for multiple *R*-squared (*F* tests, ***P < 1e-04). bio1, Annual Mean Temperature; bio2, Mean Diurnal Range; bio3, Isothermality; bio4, Temperature Seasonality; bio5, Max Temperature of Warmest Month; bio6, Min Temperature of Coldest Month; bio7, Temperature Annual Range; bio8, Mean Temperature of Wettest Quarter; bio9, Mean Temperature of Driest Quarter; bio10, Mean Temperature of Warmest Quarter; bio11, Mean Temperature of Coldest Quarter; bio12, Annual Precipitation; bio13, Precipitation of Wettest Month; bio14, Precipitation of Driest Month; bio15, Precipitation Seasonality; bio16, Precipitation of Wettest Quarter; bio17, Precipitation of Driest Quarter; bio18, Precipitation of Warmest Quarter; bio19, Precipitation of Coldest Quarter.

To complement the phylogenetic analyses, we performed genetic clustering using sNMF in the R package LEA (Frichot et al. 2014; Frichot and François 2015). After filtering missing data and SNPs showing evidence of linkage disequilibrium from the initial matrix of 54,634 SNPs, population genomic analyses in sNMF provided support for *K *=* *5 genetic groups (fig. 2*b* and *c*; supplementary figs. S3 and S4, Supplementary Material online) based on the cross-entropy criterion. Results were similar to the phylogenetic analyses, showing strong evidence for the western Desert (DST) cluster with strong geographic structure, a small Southern (STH) population and a third larger Plains (PLN) population consisting of three subpopulations (Plains South, Plains Central, Plains North), with substantial shared ancestry amongst them (fig. 2). We chose to treat the data as three populations for demographic modeling rather than five to focus on the deepest divergences from the phylogenetic analysis. Further, the additional structure detected with *K *=* *5 likely represented isolation by distance (IBD; see below). The major split between two groups separating the western (DST) and eastern (STH+PLN) populations (*K *=* *2) was recovered in virtually all analyses, and runs with the lowest cross-entropy levels supported the partition shown in figure 2. For all demographic modeling (i.e., Bayesian phylogenetics and phylogeography [BPP], MOMENTS), we defined two sets of analyses on a reduced subset of individuals, one including sample KK104 (admixed) and one without (nonadmixed). We focused on this individual for several reasons: 1) it was the only sample included in the analyses where <50% of its genome traced back to a single ancestral population (fig. 2*b*); 2) the genomic background for the individual spanned two divergent lineages (fig. 2); 3) this individual was placed in a mixed STH+PLN lineage based on the mtDNA data (supplementary fig. S2, Supplementary Material online). These results were likely because the individual was captured near the boundary of two lineages (see Discussion for additional information). For all analyses, we compared models and parameter estimates with quantify the impact of this individual on the results.

Pairwise *F*_st_ and Nei’s genetic distance estimates supported the split between the two groups inferred from the phylogenetic and sNMF analyses, separating the western (DST) and eastern populations (STH+PLN). Both *F*_st_ and genetic distance were higher between western and eastern populations than between the two eastern populations (supplementary table S2, Supplementary Material online). Genetic distance within populations was higher among eastern populations than the western population (supplementary table S2, Supplementary Material online). Analysis of spatial genetic structure revealed a significant pattern of IBD (*P* < 0.001; supplementary fig. S5, Supplementary Material online).

Our next objective was to test for a statistical association between SNPs and environmental gradients (GEA), which can provide evidence that these lizards may be adapting to divergent climatic conditions. Correlations between SNPs and environmental variables was first performed through redundancy analysis (RDA) using the R package vegan. Our global model and first of two redundancy axes were significant (*P* < 0.05). The global model had an adjusted *R*^2^ of 0.017. RDA identified 29 outlier SNPs based on locus scores that were ±2.5 SD, eight associated with mean temperature of the driest quarter and 21 associated with precipitation seasonality (fig. 3*a*). Individuals from our Desert population showed a positive relationship with BIO15: precipitation seasonality, and individuals in our Central Plains subpopulation exhibited a negative relationship with BIO9: mean temperature of the driest quarter (fig. 3*b*).

We also used latent factor mixed models (LFMM) (Frichot et al. 2013; Frichot and François 2015; Caye et al. 2019) to statistically correlate SNPs among 5,560 loci with environmental gradients, after controlling for population structure (supplementary fig. S6, Supplementary Material online). The importance of bioclimatic gradients was evaluated by computing a multiple-squared correlation between each variable and the SNPs detected by LFMM for that variable. The most important bioclimatic variables for association with allele frequencies were BIO9: mean temperature of driest quarter (correlated with 95 loci, *R*-squared=0.78, *P*=1.40e-09), BIO17: precipitation of driest quarter (correlated with 117 loci, *R*-squared=0.82, *P*=1.26e-05), BIO15: precipitation seasonality (correlated with 53 loci, *R*-squared=0.86, *P*=1.98e-17), BIO19: precipitation of coldest quarter (correlated with 54 loci, *R*-squared=0.66, *P*=5.23e-08), and BIO2: mean diurnal range (correlated with ten loci, *R*-squared=0.42, *P*=3.7e-06, fig. 3*c*). The high congruence between RDA and LFMM indicated that drought-related variables were important in shaping genomic variation in the species.

### Historical Demography under the MSC Model

Bayesian phylogenetics and phylogeography (Yang 2015; Flouri et al. 2018) was run for three purposes: to provide additional evidence for divergence among the three primary lineages (analysis A11), to estimate a species tree (analysis A01), and to estimate divergence times and effective population sizes (analysis A00). A11 analysis (species tree estimation and species delimitation) of both our admixed and nonadmixed data resulted in posterior probabilities of approximately 1.0 for each of the three populations (DST, STH, PLN). All species tree analyses placed the STH and PLN as sister with a posterior probability of 1.0. Effective population size (*N*_e_) estimates from the A00 analysis showed signs of both population growth and decline following divergence (supplementary table S3, Supplementary Material online). In comparing the *N*_e_ estimates for runs containing KK104 and runs without, six of the seven parameters overlapped within the 95% highest probability density (HPDs). The results differed most in their estimates for our DST (pop 1) population (admixed=348,125 vs. nonadmixed=233,593) as well as the most recent common ancestor of our ingroup (admixed=772,968 vs. nonadmixed=625,781). To minimize potential biases in parameter estimation, the following *N*_e_ values were from runs with KK104 removed. Our ingroup most recent common ancestor showed an *N*_e_ of approximately 625k with the descendant populations having *N*_e_ values of approximately 233k for DST and approximately 930k for the combined STH+PLN population. After the split of the STH+PLN populations, there was a reduction in *N*_e_ to STH (∼575k) and PLN (∼157k). These results are consistent with peripheral population expansion following divergence.

**Fig. 4.**
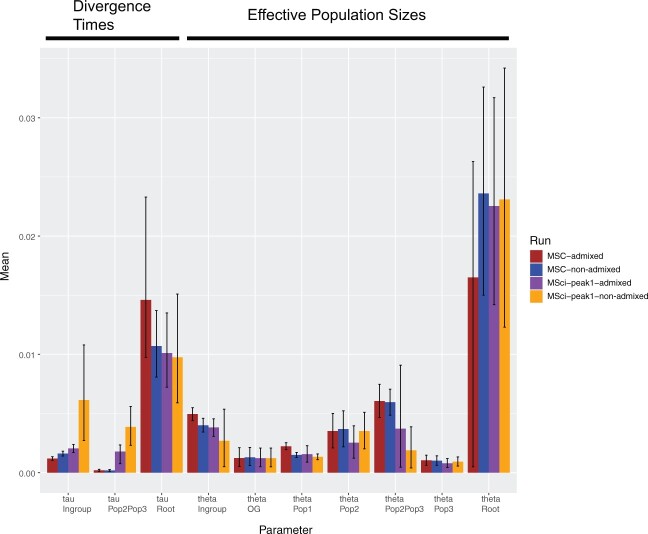
Comparison of parameter estimates from multispecies coalescent (MSC) analysis in BPP (analysis A00) with (brown) and without (blue) the highly admixed/outlier individual from Pop1 (KK104). Purple bars depict parameter estimates based on the MSci model from the data including KK104, whereas orange bars represent MSci estimates without KK104. Error bars represent 95% HPDs. Pop1, Desert (DST); Pop2, Southern (STH); Pop3, Plains (PLN); OG, outgroup (*Phrynosoma solare*).

In addition to potential bias in *N*_e_ estimates due to admixture or mixed ancestry, we found evidence for biases in divergence times (fig. 4). Including sample KK104 resulted in an older divergence time at the root while the divergence time of the ingroup was younger. Again, to minimize any biases regarding interpretation, we focused on the results with this sample removed. Assuming a divergence time of 20 Ma for *P. cornutum* and *P. solare* (Leaché and Linkem 2015) resulted in an estimated substitution rate of 0.000535 substitutions per site per million years, similar to the previously estimated mean genome-wide rate for lizards of 0.0008 by Perry et al. (2018). Thus, independent data supported a relatively slow rate of substitution, compared with faster rates found in other studies (Green et al. 2014; Tollis et al. 2018). Basing our calibration on a rate of 0.0008 substitutions per site per million years, divergence times for both nodes fell clearly in the Quaternary (supplementary fig. S7, Supplementary Material online). Combining these results with the divergence estimates from the mtDNA in BEAST (initial divergence of 5 Ma), a late Pliocene-early Pleistocene divergence event appears to be a likely scenario for the initial split.

### Demographic Models

**Fig. 5.**
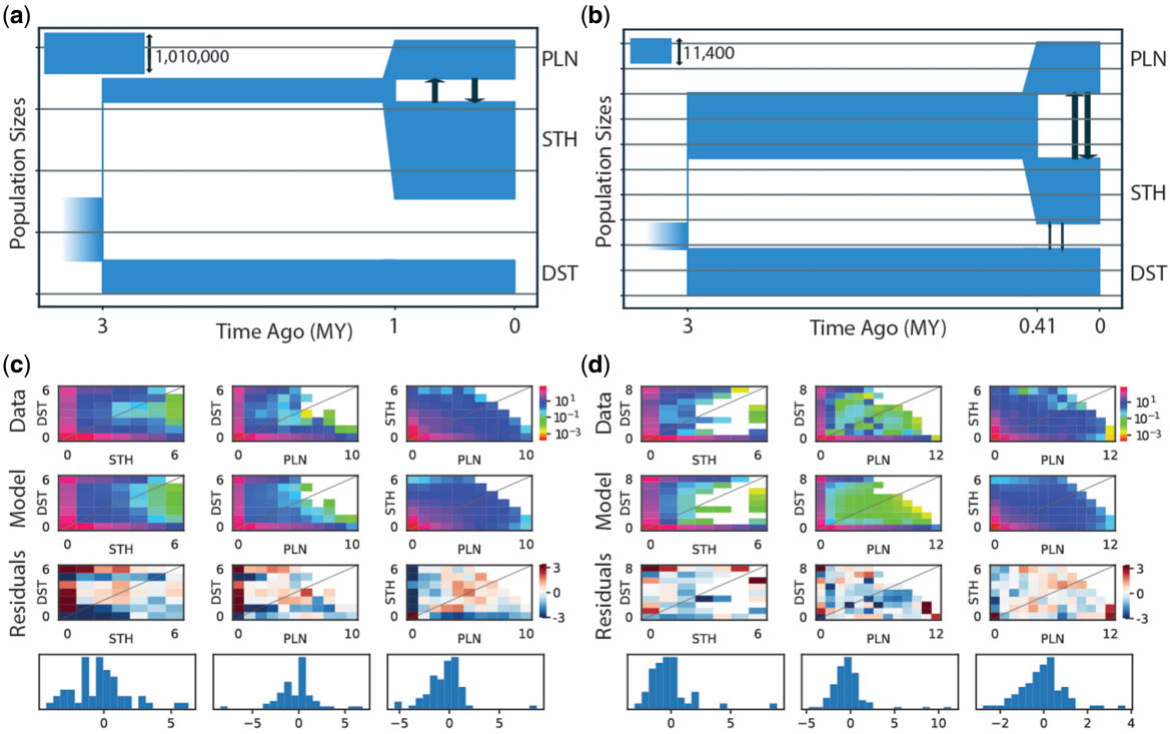
(*a*, *b*) The demographic model selected from the program MOMENTS for the *Phrynosoma cornutum* populations using the 3D-site frequency spectrum (3D-SFS) for the Admix (*a*) and NoAdmix (*b*) data sets. The reference population (*N*_ref_) was calculated from estimates of theta produced during demographic modeling (theta=4 *N*_ref_*μ*; see supplementary table S4, Supplementary Material online), where *μ* is the substitution rate which was set to 0.0008 substitutions per site per million year. (*c*, *d*) The fits between the 3D-SFS model and the data with the resulting residuals (positive residuals indicate that the model predicted too many SNPs in that entry).

**Fig. 6.**
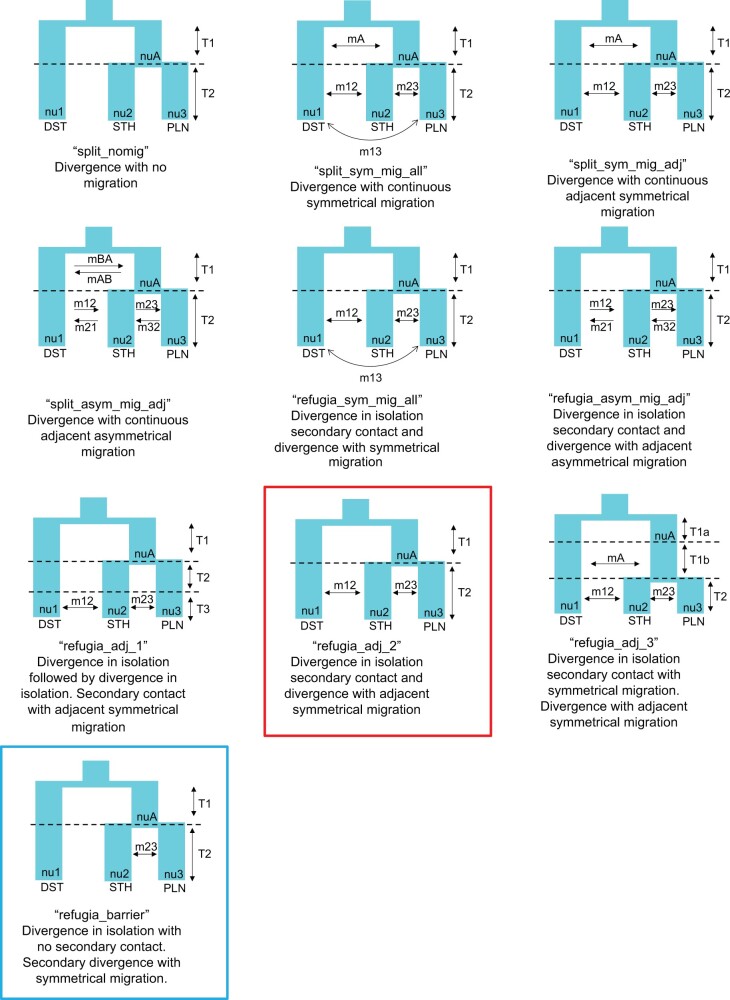
Demographic models explored using the program MOMENTS. Analyses were performed with and without sample KK104 that had substantial mixed ancestry. The data set with KK104 favored the “refugia_barrier” model (blue), whereas the data set without KK104 supported the “refugia_adj_2” model (red).

Our MOMENTS (Jouganous et al. 2017) analyses were used to test three hypotheses regarding historical divergence: allopatric divergence due to the Rio Grande, divergence due to paleoclimate change, and divergence due to ecological gradients. Each hypothesis makes assumptions regarding the importance of gene flow during evolutionary history (Leaché et al. 2019). For consistency with the BPP analyses, we analyzed the same set of individuals. The top ranked models were similar across the two data sets (with and without the admixed sample KK104), consisting of an initial split between DST and the ancestral population of STH and PLN, followed by a period of no gene flow before final diversification between STH and PLN populations with gene flow (fig. 5 and supplementary table S4, Supplementary Material online). The data set including KK104 suggested that gene flow only occurs between the STH and PLN populations. In contrast, the data set that did not include the admixed individual suggests that there was gene flow between DST and STH and between STH and PLN populations. We were unable to perform likelihood ratio tests for the data set without the admixed individual due to our top two models being unnested. Likelihood ratio tests for the data set including the admixed individual failed to reject the nested model suggesting a barrier to gene flow when compared with the model favored by the other data set, therefore it was considered the best model for the admixed data set (*D*_adj_=−2,515.84; *P*=1). AIC weights for the admixed data set strongly supported the refugia_barrier model (0.9980), whereas the nonadmixed data set favored the refugia_adj_2 model (wAIC=0.7328; table 1; fig. 6). However, the refugia_barrier model was within the 95% confidence interval for the no admixture data set.

**Table 1:** **AIC, ΔAIC, Relative Likelihood, and Weighted AIC (wAIC) Calculations for Each Demographic Model Considered (see** **fig. 6** **) for Each Data Set (Upper Panel, Nonadmixed; Lower Panel, Admixed) in the Program MOMENTS**

Model	AIC	ΔAIC	Relative L	wAIC
Nonadmixed data set				
refugia_adj_2	514.28	0.00	1.00	0.73
refugia_asymmig_adjacent	517.90	3.62	0.16	0.12
refugia_barrier	518.48	4.20	0.12	0.09
split_nomig	519.38	5.10	0.08	0.06
refugia_adj_1	529.54	15.26	0.00	0.00
refugia_adj_3	553.04	38.76	0.00	0.00
split_asymmig_adjacent	559.26	44.98	0.00	0.00
split_sym_mig_all	600.70	86.42	0.00	0.00
split_symmig_adjacent	624.66	110.38	0.00	0.00
refugia_symmig_all	629.98	115.70	0.00	0.00
Admixed data set				
refugia_barrier	738.38	0.00	1.00	1.00
refugia_adj_2	750.78	12.40	0.00	0.00
refugia_adj_1	778.82	40.44	0.00	0.00
refugia_adj_3	779.32	40.94	0.00	0.00
split_asymmig_adjacent	799.04	60.66	0.00	0.00
split_nomig	840.36	101.98	0.00	0.00
split_sym_mig_all	870.88	132.50	0.00	0.00
refugia_asymmig_adjacent	884.50	146.12	0.00	0.00
split_symmig_adjacent	992.34	253.96	0.00	0.00
refugia_symmig_all	1,226.80	488.42	0.00	0.00

Note.—Nonadmixed, without KK104; admixed, with KK104.

### Accommodating Gene Flow under the MSci Model

**Fig. 7.**
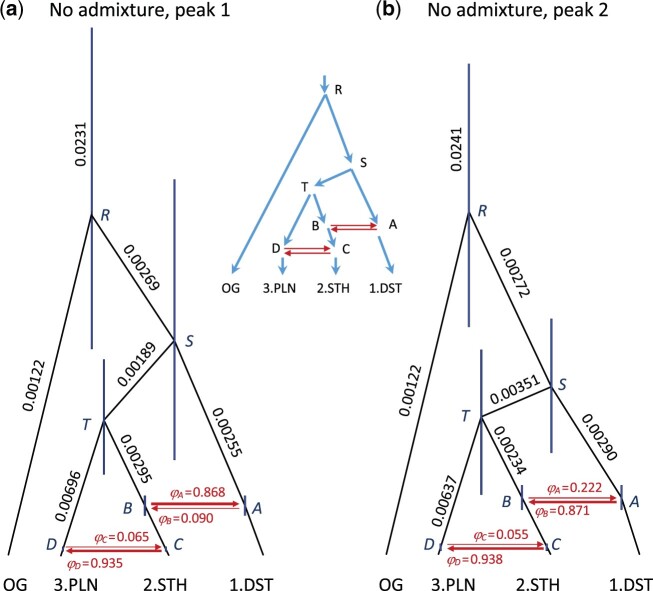
Two local peaks in the posterior for parameters in the MSci model in the BPP analysis of the data without the admixed sample KK104. The two peaks represent two hypotheses that have nearly equal support from the data, due to the species tree being nearly a trichotomy. Posterior means of node ages (*τ*s) are used to draw branches, and the node bars represent the 95% HPD credibility intervals (CIs). Numbers next to branches are posterior means of population sizes (*θ*s) (see table 2); not all population sizes are shown. The model assumes two BDI events (*A*↔*B* and *C*↔*D*), and the thickness of the horizontal branches indicates the estimated introgression probability (*φ*). According to the first peak (*a*), the lineage A-DST comprised *φ*_A_=86.8% migrants from lineage *TB* and 1−*φ*_A_=13.2% from lineage *SA*. In contrast, the second peak (*b*) suggests that the lineage *A*-DST is 22.2% from lineage *STB* and 77.8% from lineage *SA*. Estimates of *φ*s at the other three nodes (*B*, *C*, and *D*; see table 2) are interpreted in the same way. The phylogenetic network in the center represents the model specified in BPP.

Although the MSC model can accommodate coalescent stochasticity due to ILS, it explicitly assumes no gene flow once populations diverge. This assumption is likely violated in many systems, particularly in analyses of closely related species or populations. Thus, we performed a series of analyses under the MSC-with-introgression (MSci) model in BPP (Flouri et al. 2020) to compare demographic parameter estimates from the MSC analyses. We again analyzed both the admixed (with sample KK104) and nonadmixed (without sample KK104) data sets (500 loci in each case). In each data set, there were two local peaks in the posterior distribution, which corresponded to two sets of parameter values and may be considered two demographic hypotheses (fig. 7 and supplementary fig. S8, Supplementary Material online; table 2). The two peaks fit the data nearly equally well because the species tree is close to a trichotomy with two divergence times close to each other. For the admixed data, the Markov chain Monte Carlo (MCMC) run often visited only one peak. For the nonadmixed data, the MCMC run jumped between the peaks, with introgression probabilities *φ*_A_ and *φ*_B_ showing bimodal distributions. Note that the introgression probability *φ*_A_ is the proportion of population *A* composed of migrants from population *TB*, whereas 1−*φ*_A_ is the contribution from population *SA* (fig. 7). In other words, when we trace the genealogical history of sequences sampled from modern species/populations backward in time and reach node *A*, each sequence will take the two parental paths *BT* and *AS* with probabilities *φ*_A_ and 1−*φ*_A_, respectively. We separated the samples for the two peaks depending on whether *φ*_A_>½. Peak 1 (with *φ*_A_>½) consisted of approximately 86% of the MCMC samples. The subsamples corresponding to the same peak were noted to be similar between runs and those from different runs were combined to produce the posterior summary for that peak (table 2).

**Table 2:** **Posterior Means and 95% HPD CIs (in Parentheses) of Parameters in the Introgression (MSci) Model of** **Figure 7** **Obtained from BPP Analyses of Data That Either Include or Exclude the Admixed Sample KK104**

Parameter	Nonadmixed Data without KK104		Admixed Data with KK104	
	Peak 1 (*φ*_A_>½)	Peak 2 (*φ*_A_<½)	Peak 1	Peak 2
*θ*_OG_	1.22 (0.49, 2.08)	1.22 (0.51, 2.08)	1.22 (0.50, 2.08)	1.23 (0.49, 2.09)
*θ*_DST_	1.34 (1.10, 1.58)	1.34 (1.09, 1.59)	1.56 (0.89, 2.28)	1.52 (0.88, 2.25)
*θ*_STH_	3.52 (2.02, 5.10)	3.54 (1.87, 5.25)	2.53 (1.22, 3.96)	2.50 (1.29, 3.93)
*θ*_PLN_	0.93 (0.56, 1.33)	0.92 (0.51, 1.43)	0.79 (0.44, 1.19)	0.73 (0.41, 1.14)
*θ*_R_	23.1 (12.3, 34.2)	24.1 (15.6, 33.5)	22.5 (14.2, 31.7)	22.6 (14.2, 31.9)
*θ*_S_	2.69 (0.50, 5.37)	2.72 (0.75, 4.45)	3.82 (3.07, 4.55)	3.72 (3.01, 4.46)
*θ*_T_	1.89 (0.40, 3.88)	3.51 (0.37, 10.2)	3.72 (0.46, 9.08)	3.74 (0.37, 11.3)
*θ*_A_	**2.55 (0.34, 7.10)**	**2.90 (0.95, 4.33)**	**2.15 (1.46, 2.88)**	**3.80 (2.51, 5.15)**
*θ*_B_	**2.95 (1.06, 4.63)**	**2.34 (0.38, 6.15)**	**3.49 (1.31, 5.12)**	**2.17 (1.51, 2.93)**
*θ*_C_	4.27 (2.92, 5.65)	4.55 (3.11, 5.96)	2.10 (0.42, 4.98)	2.48 (0.45, 5.88)
*θ*_D_	6.96 (0.34, 20.2)	6.37 (0.33, 19.5)	11.56 (1.00, 26.0)	11.27 (1.01, 25.4)
*τ*_R_	9.75 (5.90, 15.1)	8.53 (5.65, 12.2)	10.11 (7.23, 13.5)	10.00 (7.21, 14.1)
*τ*_S_	6.14 (2.72, 10.8)	4.17 (2.52, 5.86)	2.05 (1.70, 2.39)	2.10 (1.78, 2.43)
*τ*_T_	3.86 (2.31, 5.60)	3.42 (1.48, 5.09)	1.78 (0.76, 2.34)	2.01 (1.62, 2.42)
*τ*_A_=*τ*_B_	1.42 (1.12, 1.70)	1.41 (1.10, 1.72)	0.18 (0.09, 0.26)	0.17 (0.10, 0.29)
*τ*_C_=*τ*_D_	0.17 (0.10, 0.24)	0.17 (0.08, 0.26)	0.13 (0.07, 0.20)	0.13 (0.07, 0.20)
*φ*_A_	**0.868 (0.668, 0.998)**	**0.222 (0.034, 0.469)**	**0.129 (0.078, 0.180)**	**0.873 (0.817, 0.923)**
*φ*_B_	**0.090 (0.004, 0.204)**	**0.871 (0.421, 1.000)**	**0.019 (0.000, 0.043)**	**0.985 (0.968, 1.000)**
*φ*_C_	0.065 (0.016, 0.126)	0.055 (0.013, 0.110)	0.250 (0.079, 0.605)	0.165 (0.069, 0.263)
*φ*_D_	0.935 (0.869, 0.991)	0.938 (0.877, 0.990)	0.817 (0.606, 0.981)	0.855 (0.752, 0.953)

Notes.—There are two local peaks in the posterior under the model for both the nonadmixed and admixed data, which differ mainly in four parameters, with *φ*′_A_ ≈ 1−*φ*_A_, *φ*′_B_ ≈ 1−*φ*_B_, *θ*′_A_ ≈ *θ*_B_, and *θ*′_B_ ≈ *θ*_A_ (highlighted in bold). MCMC samples around each peak are summarized separately. The introgression probability for any BDI event is defined for the horizontal branch: for example, *φ*_A_ is for branch BA, whereas the vertical branch SA has 1−*φ*_A_ (fig. 7). Divergence and introgression times (*τ*) are the ages of nodes on the tree. Population sizes (*θ*) correspond to branches on the tree, identified by the daughter node of the branch (e.g., *θ*_S_ is for branch RS and *θ*_A_ is for branch SA). Both *τ* and *θ* are measured in the expected number of mutations per site. OG, outgroup; DST, desert; STH, southern; PLN, plains. Estimates of *θ* and *τ* are ×1,000.

We discuss the genetic history implied by Peak 1 for the nonadmixed data, and then examine the similarities and differences of Peak 2 and of the results from the admixed data. When we trace the history of the samples backward in time, Peak 1 implies the following (fig. 7*a*). The DST sequences mostly (with probability *φ*_A_=86.8%) trace back to node *B* (or branch *TB*), before taking the path *TSR* to the root of the tree. Sequences from STH will reach node *C* and then mostly (with probability 1−*φ*_C_=93.5%) trace back to node *B*. Sequences from PLN will reach node *D* and mostly (with probability *φ*_D_=93.5%) take the *DCB* route to reach *B*. Thus, most sequences from populations STH and PLN will be in the same ancestral population *C* by the time *τ*_C_=*τ*_D_ ≈ 0.00017, whereas most sequences from DST will meet those from STH or PLN in ancestral population *B* by time *τ*_A_=*τ*_B_ ≈ 0.00141. Note that, in BPP, both divergence (or introgression) times (*τ*s) and population sizes (*θ*s) are measured in units of expected number of mutations per site.

Peak 2 for the nonadmixed data is a minor peak in the posterior (fig. 7*b*). It implies that most sequences from populations STH and PLN will be in the same ancestral population *C* at time *τ*_C_=*τ*_D_ ≈ 0.00017, whereas most sequences from DST will meet those from STH or PLN in ancestral population *A* by time *τ*_A_=*τ*_B_ ≈ 0.0014. Beyond nodes *AB*, the divergence times and population sizes on the paths to the root are similar between Peaks 1 and 2.

The two peaks for the admixed data are even more similar to each other because the inferred species tree has nearly a trichotomy with *τ*_S_ ≈ *τ*_T_, with near perfect matching of the parameters between the peaks: *φ*’_A_ ≈ 1−*φ*_A_, *φ*’_B_ ≈ 1−*φ*_B_, *θ*’_A_ ≈ *θ*_B_, and *θ*’_B_ ≈ *θ*_A_ (supplementary fig. S8, Supplementary Material online and table 2). Most sequences from populations STH and PLN meet in population *C* at time *τ*_C_=*τ*_D_ ≈ 0.00013, whereas most sequences from DST meet those from STH or PLN in population *T* at time *τ*_T_=0.00178 according to Peak 1 or in population *S* at time *τ*_S_=0.00210 according to Peak 2. Beyond nodes *S* or *T*, the divergence times and population sizes on the paths to the root are almost identical between Peaks 1 and 2. Thus, if we consider the expected coalescence times between sequences from the three populations, or if we consider similarly sequence distances between populations, the two peaks for each data set made very similar predictions.

Finally, we compared parameter estimates from the MSci model with those of the MSC model (fig. 4). The MSci model simultaneously accommodates deep coalescence and gene flow when estimating common evolutionary parameters. In general, ignoring gene flow when it is present leads to underestimation of divergence times and overestimation of population sizes. There was a relatively large effect of including/excluding sample KK104 on divergence times. Assuming a mutation rate of 0.0008, calibrated divergence times under the MSci model were 4.83 Ma for node *T* and 7.68 Ma for node *S*. Introgression times were 1.78 Ma for *τ*_A_=*τ*_B_ and 213 Ka for *τ*_C_=*τ*_D_ (see fig. 7 for node labels). We provide calibrated estimates for the nonadmixed Peak 1 data set only, as that is our best estimate of the evolutionary history of these populations.

### Species Distribution Modeling

**Fig. 8.**
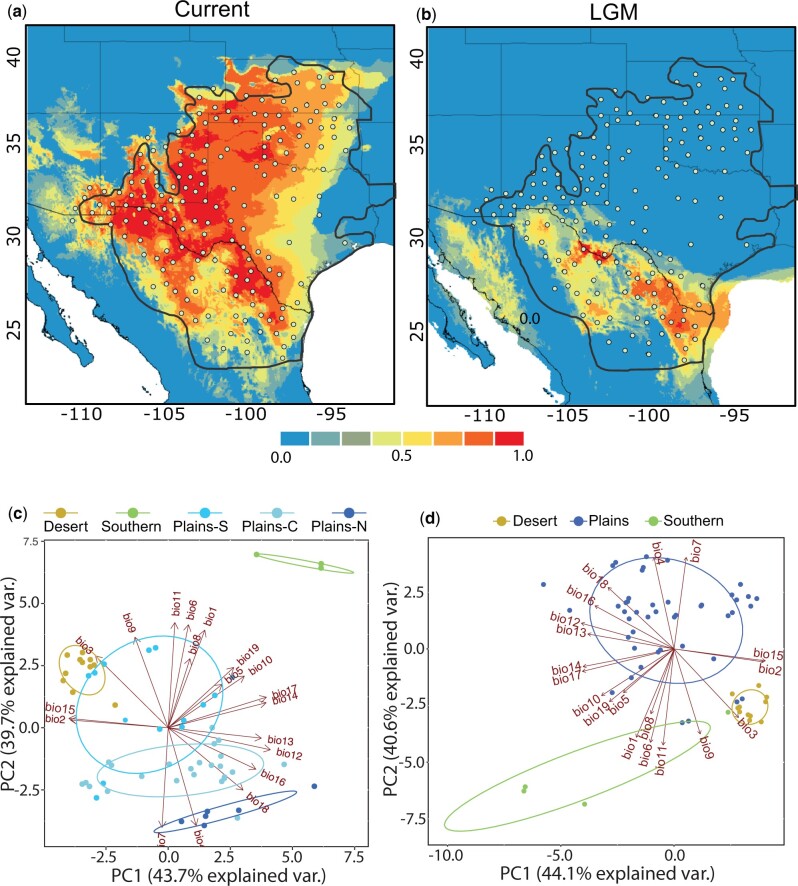
Climatic niche model for *Phrynosoma cornutum* built using the Wordclim bioclimatic variables with resolution of 2.5 min for the current climatic conditions (*a*) and projected on the MIROC and CCSM (*b*) of the LGM climate (mean of models shown). The models were visualized using logistic probability values. Warmer colors indicate a higher probability for species presence. The outer blue line shows the known range of *P. cornutum.* Dots represent the spatially filtered occurrence records used to create models. Climatic niche space occupied by each of the five genetic clusters (color-coded circles) identified in the sNMF analysis (*c*) and similar results for the primary three clusters/lineages used for demographic modeling (*d*). The first two principal components derived from 19 bioclimatic variables (arrows) of the WorldClim data set are shown.

We estimated SDMs to further test the hypothesis that lineage divergence was caused by paleoclimate change (fig. 8*a* and *b*). The SDMs estimated from the last glacial maximum (LGM) revealed niche space in northern Mexico and along the border in southern TX and NM. The eastern and central (near Big Bend) portions of this area held the highest probabilities of occurrence. The northern edge of the LGM niche space coincided with our current STH population in the east and the DST population in the central region. The models also revealed a potential disjunct niche space, albeit with lower probabilities of occurrence, between the western edge of the Chihuahuan desert to the east and the Sonoran Desert to the west (outside of the current range of the species). The current SDM shifted the suitable niche northward expanding across the plains of TX, up into CO, OK, and KS, and connecting with the expanding range in southern AZ and NM. The eastern and larger area of the current SDM occupies Level 1 Ecoregion 9 The Great Plains, whereas the western and smaller portion occurs over Ecoregion 10 North American Deserts. The PCA analysis of the climatic niche space occupied by our genetic clusters showed the greatest dissimilarity between the areas occupied by our DST and STH populations with no overlap on the PC1 axis (fig. 8*c*). The climate niche space occupied by our three PLN subpopulations showed the greatest similarity and considerable overlap on the PC1 axis. All PCAs indicate that the three main lineages/populations inhabit a substantially different niche space (fig. 8*d*).

## Discussion

### Genetic Structure and Demography

Speciation occurs when barriers to gene flow arise and separate populations. Barriers can come in the form of hard geographical divides such as mountains and rivers, or soft divides where the barriers to gene flow are environmental factors. Recent studies have shown these soft ecological divides may have a greater impact on diversification and speciation than the traditional hard allopatric geographical barriers (Moen and Wiens 2017; Castro-Insua et al. 2018; Myers et al. 2019). The evolutionary history of *P. cornutum* appears to further the evidence for the importance of both hard and soft allopatry in shaping species and highlight the diverse history of populations across a species range.

We found similar population structure to Williams et al. (2019) with high divergence between a desert (DST), southern (STH), and plains (PLN) clades at nuclear SNPs that correspond, respectively, to the western, southern, and northern, populations in the earlier study. By incorporating analysis of SNP data in addition to mtDNA, we were able to expand upon this earlier study by estimating divergence times between these groupings and elucidating the current and historic environmental factors that have influenced population structure. Divergence time estimates from both the mitochondrial and nuclear data (under the MSC model) suggest that *P. cornutum* populations initially diverged during the late Pliocene or early Pleistocene in the range of 2.5–3 Ma, supporting our hypothesis of cryptic diversity within the species. We arrive at this time interval based on multiple analyses of the nuclear data while taking into account the divergence estimates from our mtDNA analysis (∼5 Ma). Given the likelihood of over estimating divergence times from mtDNA due to substitution saturation owing to a quicker mtDNA mutation rate (Zheng et al. 2011), we focus predominantly on the nuclear estimates. However, we do recognize the present challenges of adopting nuclear genome-wide substitution rates. Importantly, our divergence times correlate with the onset of full scale North American glaciations (Zachos et al. 2001), which resulted in cooler and more arid conditions throughout much of the American Tropics and may also have facilitated the Great American Biotic Interchange in mammals (Bacon et al. 2016). However, our SDMs suggest that our study area in particular experienced cooler and wetter conditions, at least during the LGM.

The two primary lineages (DST, STH+PLN) may have roughly coincided geographically within refugial habitats that originated during the Pleistocene, in the Chihuahuan Desert to the east, and the Sonoran Desert to the west (figs. 1 and 7). This deep divide may be the result of niche conservatism (Wiens and Graham 2005), where these populations tracked habitats amidst a changing climate resulting in subsequent isolation, consistent with a refugial speciation model (Moritz et al. 2000). The finding of suitable habitat throughout the Sonoran Desert during the Pleistocene is noteworthy, as the current range of *P. cornutum* does not extend this far west. These historical patterns also appear congruent to those of other reptile taxa inhabiting the region, which also support a model of divergence in allopatry during the Pleistocene followed by secondary contact and gene flow (Schield et al. 2015, 2018, 2019). An alternative hypothesis for the initial split is that the Plio-Pleistocene Lake Cabeza de Vaca in the northern Chihuahuan Desert served as a biogeographic barrier leading to vicariance (Rosenthal and Forstner 2014). Unfortunately, the results of our demographic modeling make it difficult to disentangle vicariance due to paleoclimate versus the lake, as both hypotheses predict initial divergence in allopatry followed by secondary contact and gene flow. From our nuclear data, we show evidence of a second split occurring more recently in the eastern population as it expanded its range northward in response to a shifting climate opening up greater niche space as glaciation receded. It is these fluctuating Pleistocene climatic cycles driving habitat contraction and expansion that are likely to have initially shaped the current population structure and set the groundwork for further divergence.

As a population expands its range through a series of founder events, the signatures of this expansion should be evident in a reduction of population size and genetic diversity in the populations occupying the new territory (Excoffier et al. 2009). This decrease in heterozygosity at the forefront of the expansion has been illustrated in many studies of wide-ranging species (Peter and Slatkin 2013; Jezkova et al. 2016; Garcia-Elfring et al. 2017). This same signature of expansion is readily visible across our analyses. Consistent with this signature of expansion at nuclear loci, there is higher mtDNA haplotype diversity in the STH (south) population than the PLN (north) population which also suggests the expansion occurred from the south into more northern areas (Williams et al. 2019). Although our PLN population occupies by far the largest geographical area, stretching from TX to KS, it appears to have the smallest population size. Our BPP analyses indicate a reduction in *N*_e_ after the STH and PLN populations diverged, furthering the evidence for this northward expansion originating from the south. Interestingly, evidence from our population structure analysis indicates that members of this expanding PLN population do share ancestral genetic variation with our DST population. The existence of some highly admixed individuals (KK104, 7R10L) supports our demographic results and point toward secondary contact and gene flow post divergence. Taken together, these results suggest that climatic cycling during the Pleistocene was the most likely catalyst for range expansion and secondary contact. An alternative hypothesis for admixture may be due to human mediated movement of *P. cornutum* owing to its popularity as a pet and symbol of the American Southwest. Other studies have shown evidence of translocations with admixed individuals appearing far removed from boundary areas (Williams et al. 2019). This human-mediated movement may play a role in the mitochondrial introgression. It may also provide the reason the Rio Grande does not appear to be an insurmountable barrier to gene flow between the populations. However, we note that signals of introgression and admixture are restricted to the periphery of the range of each lineage. For example, sample KK104 was collected in Brewster Co., TX, which is substantially farther east than other individuals in the clade and in close geographic proximity to samples encompassing our PLN population. This sample is also nested in the PLN+STH mtDNA lineage and not the DST lineage, indicating introgression. Similar geographic patterns are also found with sample 7R10L from Dimmit/La Salle County, TX. A previous study with denser sampling in western TX, found that the DST (western) population extended from El Paso Co. to Brewster Co. (Williams et al. 2019), on the opposite side of the Rio Grande. Admixture between the western and eastern groups was concentrated in Jeff Davis and Brewster Counties, although as previously mentioned, there were some admixed individuals that were far removed from this potential boundary area (Williams et al. 2019). More comprehensive sampling throughout TX, particularly near contact zones, is required to determine the precise locations of lineage boundaries.

The Riverine Barrier hypothesis would suggest that the Rio Grande could act as a vicariant barrier to gene flow, isolating the groups on either side and shaping the population structure (Pellegrino et al. 2005; Lanna et al. 2020). Geographically, the river does appear to divide the populations (fig. 1) with only three individuals from our DST population appearing on the eastern side of the river. It is possible that the river continues to serve as a moderate barrier to dispersal, and future studies should focus on obtaining samples from Mexico to test this hypothesis further. The demographic models we tested in MOMENTS supported different models depending on whether sample KK104 was included in the analysis. Models without KK104 (nonadmixed data set) favored secondary contact with gene flow between the populations (i.e., the refugia_adj_2 model), though the model with an explicit barrier between populations (with no gene flow to/from DST) was within the 95% CI of AIC weights. The best demographic model that included KK104 (admixed data set) was the refugia_river_barrier model (wAIC=1.0), that predicted gene flow only between the STH and PLN populations. These results highlight the importance of sampling scheme (even a single highly admixed individual) for demographic inference, and further studies are needed to explore this phenomenon more closely. The presence of a heavily admixed specimen from the DST population (KK104) from the eastern side of the river, along with DST ancestral genetic variation appearing in individuals throughout the range suggests that the river is not an absolute barrier. The importance of rivers as vicariant barriers to gene flow has come under recent scrutiny with studies showing they may not provide the impasse once thought, with one study finding them noneffective in 99% of Amazonian species studied (Nazareno et al. 2017; Santorelli et al. 2018; Lanna et al. 2020). Again, it seems best to not approach this question as an all or nothing proposition as the river’s width was correlated with the strength as a barrier to gene flow (Nazareno et al. 2017). Thus, it is possible that the reduced gene flow between these populations is at least partly due to the Rio Grande. Additional sampling throughout Mexico will likely result in more power to test the efficacy of the Rio Grande as a barrier to gene flow. We also note that the Sacramento Mountains in southern NM may serve as a contemporary barrier to gene flow.

Niche divergence resulting from ecological gradients across the species’ range may play a significant role in driving continued divergence in *P. cornutum*. Among ecological gradients, precipitation is considered a major factor in furthering diversity and determining a species’ range (Hawkins et al. 2003; Wiens et al. 2013). The family Phrynosomatidae has historically existed in arid environments, with those currently occupying more mesic habitats being recently derived (Wiens et al. 2013). This historic trend highlights a family-wide pattern of migration (=recent colonization) toward areas of greater precipitation. Across the range of *P. cornutum* there exists a significant precipitation gradient, ranging from under 10 in (25.4 cm) of average annual rainfall in the western desert to over 50 in (127 cm) in the eastern reaches of the Great Plains (Pittman et al. 2007). Variables concerning precipitation account for our top three results from LFMM analysis. Further, 21 of 29 SNPs identified through RDA were associated with seasonal precipitation. Thus, we cannot refute the hypothesis that the varied levels of precipitation from across the range of *P. cornutum* are causing adaptive divergence in this system. Because of the species’ low vagility and extensive range, adaptations that prove advantageous may become fixed in the population with greater speed, compounding the effects of niche divergence (Ujvari et al. 2008). Considering morphological adaptations to arid environments are visible in the form of the interscalar channels *P. cornutum* uses to harvest rainwater (Sherbrooke 1990), it would be interesting to see if morphological variation along precipitation gradients exists among the three populations.

There are additional populations of *P. cornutum* that reside in the southeastern United States, having been introduced in the 1920s as a form of pest control. These populations already show significant morphological differences from their west coast counterparts (Heuring et al. 2019) despite the short term of geographical separation. Although it is not clear if the differences are the result of genetic drift or adaptation to unique environments, it does highlight the rapidity with which significant morphological changes can arise between populations. With the deep divergence between our DST and STH+PLN populations occurring >3 Ma, not only does it vastly increase the time frame for adaptation and further divergence to occur, it places it amongst other speciation events seen in the genus. According to a recent time-calibrated phylogeny of *Phrynosoma* (Leaché and Linkem 2015), several species pairs diverged more recently than 5 Ma, with the *P. platyrhinos–P. goodei* split occurring concurrently with our DST and STH+PLNS divergence at approximately 3 Ma. Currently, *P. cornutum* is the second oldest lineage of the genus at 20 Ma, younger than only *Phrynosomaasio*. In addition, recent genomic data (ddRADseq) suggest that *P. cornutum*, along with *P. asio* and *P. solare*, exhibits substantial genetic divergence among populations (Leaché et al. 2021). If there indeed does exist a cryptic species or subspecies within *P. cornutum*, further examination of both morphological and behavioral differences between the populations is necessary.

### Accommodating Gene Flow in Genomic Studies

The recently developed MSci model was designed to explicitly accommodate both ILS and gene flow/introgression when estimating divergence times and effective population sizes (Flouri et al. 2020). Given the presumed ubiquity of inter- and intraspecific gene flow in natural populations, the model marks a significant advancement of the field. However, the current implementation of BPP assumes that the introgression model is specified a priori, and furthermore the program may not deal with recent hybrids when inferring ancient admixture events. Here, we compare and contrast results from several BPP analyses under the MSC and MSci models, both with and without sample KK104 (admixed and nonadmixed data sets). For many parameters the 95% HPDs overlapped, though some interesting patterns emerged. Of particular note was the much older divergence times inferred from the nonadmixed data set under the MSci model versus the other three analyses (MSC-admixed, MSC-nonadmixed, MSci-peak1-admixed). The former analyses estimated divergence times of 7.68 and 4.83 Ma, whereas in the remaining analyses divergence times occurred during the Pleistocene. The admixed data also produced a much smaller introgression time *τ*_A_=*τ*_B_ than the nonadmixed data (posterior means 0.000178 vs. 0.00142; table 2). Other divergence times (such as *τ*_C_=*τ*_D_, which is assumed to be smaller than *τ*_A_=*τ*_B_, and *τ*_T_ and *τ*_S_) were also affected. Similar to MOMENTS, these differences can be explained by the impact of including a recent hybrid sample (KK104). Note that sequences sampled from two modern populations cannot coalesce until they are in the same ancestral population. Let *t*_1–23_ be the smallest sequence divergence between 1.DST and 2.STH (or 3.PLN), minimized across all loci and all sequence pairs at each locus. Then *t*_1–23_>*τ*_A_. As KK104 appears to be a recent hybrid, the divergence time at some loci can be very small, and those small distances will force *τ*_A_ to be very small. Note that under the coalescent model, species divergence times and introgression times are determined mostly by the minimum, rather than the average, sequence divergence between species. The result suggests that hybrid samples should be avoided when one aims to infer ancient introgression history. Similarly, we suggest that the results from the nonadmixed data may represent a more realistic description of the history of divergences and introgressions for those lineages. We leave it to future studies to more thoroughly assess the impact of admixed samples on the estimation of divergence times under the MSC and MSci models.

As discussed above, the peaks in the posterior of figure 7 and supplementary figure S8, Supplementary Material online, are difficult to distinguish using genomic sequence data. According to the theory developed by Yang and Flouri (2021), bidirectional introgression (BDI) events generate unidentifiability issues of two types: “within-model” and “between-model,” depending on whether the species involved in the introgression are sister or nonsister species. The “within-model” unidentifiability is essentially a label switching issue as the MCMC samples parameters within a single model. We note that the two peaks in figure 7 and supplementary figure S8, Supplementary Material online, represent alternative “within-model” hypotheses that are nearly equally supported by the data. The peaks are identifiable, but very hard to distinguish with genetic data because the two speciation events occurred in quick succession (with *τ*_S_ ≈ *τ*_T_ in supplementary fig. S8, Supplementary Material online). The sequence data also provide equal support for multiple “between-model” hypotheses: the four alternative between-model hypotheses corresponding to Peak 1 for the nonadmixed data set are shown in supplementary figure S9, Supplementary Material online. These models are unidentifiable, as they make exactly the same probabilistic predictions for the gene trees and thus the same predictions for the multilocus sequence data. It is then impossible to use genomic data to distinguish such models. Researchers will need to consider additional information (e.g., habitat requirements) to help elucidate the most likely history of the species/populations. To our knowledge, this study serves as the first empirical investigation of unidentifiability issues with BDI models in BPP, and we encourage researchers interested in these models to carefully examine both classes of unidentifiability issues that may confound analysis and interpretation (Yang and Flouri 2021).

Both the MSci and isolation-with-migration models can be used with genomic data to account for gene flow when estimating divergence times and population sizes (Gronau et al. 2011; Flouri et al. 2020). The MSci model assumes periodic introgression events between species, whereas the isolation-with-migration model accommodates continuous migration rates every generation. Selecting the appropriate model for a given data set is not straightforward, and additional studies are needed to quantify the effect of model misspecification. However, our results indicate that ignoring gene flow when it is present can potentially bias parameter estimates. More specifically, divergence times are underestimated and population sizes are overestimated when gene flow is not explicitly accounted for. Interestingly, we find no effect on species delimitation or species trees. This result is most likely due to the small number of populations studied. Our results are remarkably similar to previous simulation studies that also demonstrated similar biases in parameter estimates (Leaché et al. 2014). Thus, we further advocate careful consideration of models, assumptions, and sampling regimens when estimating demographic histories from genomic data.

## Conclusions

We investigated the history of diversification within *P. cornutum* throughout the southwestern and central United States by using genomic data to examine the hard and soft allopatric forces that have shaped population genetic structure. We find evidence for an initial divergence during the Plio-Pleistocene (possibly the Miocene) that was likely driven by habitat fragmentation due to climate fluctuations, vicariance due to the Rio Grande, and potentially Lake Cabeza de Vaca, followed by a subsequent northward range expansion as the receding glaciation opened up novel habitats. This expansion facilitated divergence along sharp environmental clines and possible adaptation to a divergent niche space. Whether the population-level diversity uncovered through this study rises to the level of species will require further investigation (e.g., estimation of hybridization rates in contact zones for comparison with the long-term introgression rate), additional data (i.e., morphology), and dense population sampling, especially throughout Mexico. The evolutionary history presented here highlights the importance of both hard and soft allopatric forces in shaping a species through gene flow, as the lineage divergences appear at least partially influenced by a changing habitat and environmental niche. Finally, this study should serve as a foundation for the exploration of powerful new models of demographic inference that make use of genomic data sets.

## Materials and Methods

### Sampling and Data Collection

Tissue samples (75) of *P. cornutum* were obtained from both museum specimens and field samples collected from multiple sites throughout KS, OK, CO, NM, TX, and AZ (fig. 1 and supplementary table S1, Supplementary Material online). A single *P. solare* individual from Pima County, AZ was also included as an outgroup taxon. All new collections were approved by the IACUC Committee at Miami University (protocol number 992_2021_Apr).

Genomic DNA was extracted from liver or muscle tissue using the Qiagen DNeasy Blood & Tissue Kit (Hilden, Germany) following manufacturer protocols. DNA quantity and quality were measured on a NanoDrop spectrophotometer. Aliquots of DNA extracts were shipped to LGC Genomics (Berlin, Germany) for library prep and sequencing using a modified GBS (Elshire et al. 2011; Arvidsson et al. 2016) approach. The technique, termed nGBS digests genomic DNA using the *Ms*II restriction enzyme and utilizes a subsequent normalization step after adapter ligation to remove fragments with a high number of copies. The method is particularly suited for species lacking a reference genome. Size-selected fragments were QC-ed and sequenced on an Illumina NextSeq flow cell (150 bp PE). Data were demultiplexed using Illumina bcl2fastq v. 2.17.1.14. Two samples (FHSM16593, FHSM16898) were excluded from further analysis due to a low number of reads. All nGBS data were uploaded to the SRA (BioProjectID = PRJNA780191).

The raw, demultiplexed data were processed using ipyrad v. 0.7.30 (Eaton and Overcast 2020). The demultiplexed data were first quality filtered to remove residual adapter sequences (using *cutadapt*) and low-quality bases. Reads were then clustered within and between individuals based on 85% similarity, which is the default value recommended by the program authors. A minimum of 30 individuals per locus (∼39% of samples) was required to keep loci in the final assembly, resulting in a concatenated matrix of approximately 8 million base pairs and 57,459 retained loci. Default values were also used for the remaining parameters. We also performed additional assemblies using a clustering threshold of 90%, and the results were qualitatively similar.

We obtained new mtDNA sequences from all samples to compare with the GBS data. Approximately 1,400 bp of mtDNA were collected from each sample, encompassing the entire *ND1* gene, *tRNA leucine*, *tRNA isoleucine*, *tRNA glutamine*, and portions of *16S* and *tRNA methionine*. PCR amplification was performed using previously published primers (Leaché and McGuire 2006) and the *Taq* PCR kit (New England Biolabs, Ipswich, MA). Reactions (25 μl) consisted of the following: 2.5 μl 10× reaction buffer, 0.5 μl 10 mM dNTPs, 0.5 μl 10 μM forward primer (16dR), 0.5 μl 10 μM reverse primer (tMet), 0.125 μl *taq* DNA polymerase, 19.875 μl ddH_2_O, 1 μl template DNA. All PCRs were performed on a BIO-RAD T100 Thermal Cycler using the following cycling conditions: initial denaturation at 95 °C (30 s), 30 cycles of denaturation at 95 °C (30 s), annealing at 55 °C (1 min), and extension at 72 °C (1 min), followed by a final extension at 72 °C for 5 min and samples held indefinitely at 4 °C. Horizontal agarose gel electrophoresis (1%) was used to assess the success of reactions. Amplicons were enzymatically purified using ExoSAP-IT (ThermoFisher Scientific, Waltham, MA) following manufacturer’s recommendations. Purified products were sent to GENEWIZ (South Plainfield, NJ) for Sanger sequencing. Due to the large fragment size, amplicons were sequenced in both directions. Raw sequence data were edited in FinchTV v. 1.5.0 (Geospiza, Inc.). Aliview v. 1.26 (Larsson 2014) was used to form contigs and perform multiple sequence alignment using Muscle (Edgar 2004). All new mtDNA sequences were deposited to GenBank (OL549193 - OL549266).

### Phylogenetic Analysis

All phylogenetic analyses were implemented through the High-Performance Computing Center (HPCC) at The College of Staten Island (CUNY). We performed both concatenated and coalescent analyses on the genomic data, as both approaches have their strengths and weaknesses (Kubatko and Degnan 2007; Chou et al. 2015; Edwards et al. 2016) and recent empirical studies show that performing both can potentially result in novel insights (Blair et al. 2019). Concatenated ML phylogenetic analysis (unpartitioned) was implemented using the hybrid MPI/Pthreads version of RAxML-ng v. 0.8.1 (Kozlov et al. 2019). A standard nonparametric bootstrap (250 reps) and ML search was implemented under a GTRGAMMA model of nucleotide substitution. Trees were rooted using *P. solare*. We also performed 20 independent ML searches from ten distinct maximum parsimony and ten random starting trees to determine if multiple likelihood peaks were present in the data. Robinson–Foulds distances were calculated between the 20 unrooted trees. These analyses were performed using the full multilocus data versus individual SNPs.

We also performed Bayesian phylogenetic analyses in ExaBayes v. 1.5 (Aberer et al. 2014). ExaBayes is explicitly geared toward Bayesian analysis of large phylogenomic data sets generated through next-generation sequencing, utilizing MPI parallelization to increase computational efficiency. Default priors were used for all parameters. Analyses were run for 50 million generations, sampling every 5,000 generations. Mixing and effective sample sizes (target ESS >200) for all parameters was monitored in Tracer v.1.7.1 (Rambaut et al. 2018). A majority rule consensus tree was generated following a burnin of 25%. The unrooted topology was subsequently rooted using *P. solare*. Similar to the ML analyses, all ExaBayes runs used the full loci including invariable sites.

Coalescent-based phylogenetic analysis was performed using SVDquartets (Chifman and Kubatko 2014) in PAUP* v. 4.0a159 (Swofford 2001). SVDquartets is statistically consistent with the multispecies coalescent and first infers quartet relationships using site pattern frequencies and singular-value decomposition scores. The algorithm then uses QFM (Reaz et al. 2014) to assemble quartets into a full tree containing all taxa. Although SVDquartets can be used with multilocus sequence data, the method is particularly suited to large SNP data sets and has been recently used in other RADseq/GBS studies (Leaché et al. 2015; Eaton et al. 2016). We used the *.u.snps.phy* file from ipyrad for all SVDquartets analyses to minimize linkage of SNPs. All quartets were evaluated (1,150,626) and 100 nonparametric bootstrap replicates were used to assess nodal support. Trees were rooted using *P. solare*.

We used BEAST v. 2.6.3 (Bouckaert et al. 2019) to infer genealogical relationships and divergence times based on the mtDNA sequences. bModelTest v. 1.2.1 (Bouckaert and Drummond 2017) was specified as the substitution model for all analyses, which uses reversible-jump MCMC to switch between models. A constant size coalescent tree prior was used, a relaxed log normal clock (Drummond et al. 2006), and all remaining priors were left as defaults. We also ran a strict clock analysis for comparison. Analyses were temporally calibrated using a mitochondrial substitution rate previously calculated for a similarly sized lizard (Macey et al. 1999) and used in other studies of both *Phrynosoma* and other lizards (Bryson et al. 2012; Jezkova et al. 2016). However, to accommodate uncertainty in the rate, we specified a normal prior with a mean of 0.00805 substitutions per site per million years and a sigma of 0.0005. Chains were run for 40 million generations, sampling every 4,000 for a total of 10,000 states over independent runs. Mixing, ESS values (target >200) and parameter estimates were monitored in Tracer. TreeAnnotator was used to construct a maximum clade credibility tree annotating nodes using mean heights following a burnin of 10%.

### Population Structure and GEA Analyses

Population structure was analyzed using the nonnegative matrix factorization algorithm sNMF implemented in LEA v2.6.0, for which the number of genetic clusters, *K*, was evaluated from the cross-entropy criterion (Frichot et al. 2014; Frichot and François 2015). This criterion measures the amount of statistical information conveyed by a model with *K* clusters by comparing predictions of masked alleles to their true value, and detects the most significant subdivisions in the data. Like STRUCTURE (Pritchard et al. 2000), sNMF is a descriptive method, and visual inspection of the clustering results was used to investigate finer population structure for *K *=* *2–10. Before performing GEA analysis, SNPs were filtered out for loci with less than 50% missing data. The missing genotypes were then imputed using values predicted by the sNMF model (*K *=* *5). SNPs with minor allele frequency lower than 5%, and SNPs in strong linkage disequilibrium (*r*^2^ > 0.96) were removed from the data set.

We calculated pairwise *F*_st_ values (Weir and Cockerham 1984) between the three main populations inferred from both the sNMF and phylogenetic analyses using the R package hierfstat (Goudet 2005). We made the decision to treat these as three populations rather than five to focus on the both the deepest divergences from the phylogenetic analysis and the geographic structure of the populations (see Results). The analysis was run for 1,000 bootstraps using 95% confidence intervals to assess significance. Nei’s genetic distances (Nei 1978) were calculated using the R package StAMPP v 1.5.1 (Pembleton et al. 2013) to determine mean pairwise distances between populations and diversity within each population.

Spatial genetic structure was examined at an individual level using ML population effects parametrization (MLPE, Clarke et al. 2002). We compared geographic distance and genetic distance to test for evidence of IBD throughout the sampled distribution. This was implemented using the R packages nlme (Pinheiro et al. 2012) and corMLPE (https://github.com/nspope/corMLPE, last accessed December 1, 2021), with the correlation between population pairs as covariates, and Akaike weights calculated using the MuMIn package (Bartoń 2019). The outgroup taxon was excluded prior to performing these analyses.

Genome-wide associations with climatic gradients were investigated using LFMM, as implemented in the R package lfmm (Frichot et al. 2013; Frichot and François 2015; Caye et al. 2019). The number of factors in LFMMs were determined from the population structure analysis (*K *=* *5). Climate data were obtained from the WorldClim v2 database at the 2.5-min resolution (Fick and Hijmans 2017). All 19 WorldClim bioclimatic variables were tested for association with SNPs and a joint correlation analysis for all bioclimatic variables was performed. Significance values were obtained after Bonferroni correction for multiple testing. The importance of bioclimatic variables was evaluated by computing the coefficient of determination for each variable and the SNPs detected by LFMM for that variable. Statistical significance of determination coefficients was evaluated using Fisher tests. R code and associated data files to reproduce sNMF and LFMM analyses are available on figshare (see Data Availability section).

We also implemented RDA to assess correlation between SNPs and environmental variables using the R package *vegan* (Oksanen et al. 2016). RDA is a constrained ordination method that is a multivariate analog of linear regression and examines the amount of variation in one set of variables that explains variation in another set. In our case, how much genomic variation is explained by environmental predictors. RDA is a powerful method that can be used to infer selection, with low false-positive and high true positive rates (Forester et al. 2018). The approach performs a PCA on the response variables (SNP matrix) while constraining the PCA axes as linear combinations of the predictor (environmental) variables. In our analyses, environmental variables were represented by two bioclimatic variables from WorldClim v2 (Fick and Hijmans 2017): mean temperature of the driest quarter and precipitation seasonality. These variables were selected to account for major aspects of climate while avoiding autocorrelation among variables (Dormann et al. 2013). The significance of the entire model and each axis was evaluated using an ANOVA with 999 permutations. Effects of collinearity between environmental predictors were assessed using the function *vif.cca* to evaluate variance inflation factors. We then identified candidate SNPs based on locus score that were ±2.5 SD from the mean loading on all four constrained axes. We identified the environmental variables with the strongest associations with each candidate SNP using a Pearson’s correlation coefficient (*r*).

### Species Tree and Historical Demographic Analyses

We used BPP v4.1.3 (Yang 2015; Flouri et al. 2018) to perform a series of coalescent-based analyses on reduced subsets of data (individuals and loci). This is a Bayesian MCMC implementation of the multispecies coalescent model with and without introgression. The full likelihood approach applied to multilocus sequence alignments makes full use of information contained in both gene tree topologies and branch lengths. Unlike concatenation, the approach accommodates the coalescent fluctuation in genealogical history across the genome. Unlike two-step approaches, the likelihood calculation in the MCMC algorithm averages over gene trees and branch lengths at individual loci, accommodating their uncertainties (Rannala and Yang 2003, 2017; Yang and Rannala 2014; Flouri et al. 2020). Because our genetic clustering analyses indicated the possibility of admixture between some populations (see Results), one data set excluded a highly admixed individual with <50% of the genome originating from a single ancestral population (sample KK104) that was in an otherwise genetically distinct population while another included the individual. All other individuals used in analyses could trace >50% of their genome to a single cluster. Our goal was to test how inclusion of this sample might influence the estimation of common evolutionary parameters (e.g., species trees, divergence times, population sizes). Samples were assigned to one of three populations in *P. cornutum* (rooted with *P. solare*) following the results of the phylogenetic analyses (i.e., RAxML-ng, ExaBayes, SVDquartets) and clustering in sNMF. We chose to analyze three populations/lineages to represent the deepest divergences in the genealogy. We did not divide the Plains lineage into two populations due to the results of SVDquartets (see Results). However, all BPP analyses used individuals from only one of the two Plains lineages inferred by RAxML-ng and ExaBayes. For computational reasons, all analyses were run using 500 loci.

We first performed a series of A11 analyses to provide additional support that the populations defined by previous analyses might represent distinct populations or species (Yang and Rannala 2010; 2014). This analysis compares MSC models that differ in the number of species and in the species phylogeny. Each MSC model involves two sets of parameters: the species divergence times (*τ*s) and the population sizes (*θ*s). Both parameters are measured in the expected number of mutations per site. Four independent A11 analyses were run (two using algorithm 0 and two algorithm 1). The species model prior assumed uniform rooted trees, and the starting tree topology was based off the concatenated analyses. We specified an inverse gamma (IG) prior of IG(3,0.004) for population sizes (*θ*) and IG(3,0.05) for the divergence time at the root of the species tree (*τ*_0_). Runs were implemented using an initial burnin of 50,000 generations followed by sampling every five generations for 100,000 total samples. Convergence was assessed by examining consistency between runs. We then performed a series of species tree analyses in BPP (A01) using the same populations. Similar to previous analyses, runs were performed both with and without the admixed individual KK104 to quantify the potential impact of gene flow on species tree estimation. All A01 analyses used the same priors and sampling frequency as the A11 analyses. We compared the best tree and associated support values among runs. Finally, we performed multiple A00 analyses to estimate divergence times and effective population sizes (*N*_e_) on the species tree inferred from the A01 analyses. Again, analyses included or excluded sample KK104 to determine how gene flow might influence divergence times and population sizes. The parameter settings and priors were identical to the other BPP analyses, except that we used an initial burnin of 200,000 followed by sampling every 20 generations for 100,000 total samples. Mixing, convergence, and ESS values (target >200) were assessed using Tracer v1.6.0 (Rambaut et al. 2018).

There is still no general consensus of accurate nuclear genome-wide substitution rates for lizards. Estimates from the literature suggest that lizard rates, on average, are slightly faster than snakes (0.00077 vs. 0.00074 substitutions per site per million years, respectively; Perry et al. 2018). The assumptions and uncertainty about substitution rate directly translates to uncertainties about absolute divergence times, which can influence hypothesis testing. Thus, we used several sources of information to convert raw parameter estimates of θ and *τ* to units of effective number of individuals and millions of years, respectively. First, we used previous results for the divergence time (*T*) of *P. cornutum* and *P. solare* (∼20 Ma; Leaché and Linkem 2015) to obtain an empirical mutation rate (*μ*) estimate directly from the data (*μ* *=* *τ/T*). This calculation provided additional evidence either supporting or refuting previous rate hypotheses. We then compared our rate estimate to independently estimated genome-wide neutral substitution rate for lizards and squamates (Green et al. 2014; Perry et al. 2018; Tollis et al. 2018). Our analysis provided support for slower substitution rates, supporting the recent estimates of Perry et al. (2018). Thus, our final calibrations were based on a rate of 0.0008 substitutions per site per million years (8×10^−10^ substitutions per site per year). To obtain estimates of *N*_e_, we assumed a generation time of 2 years (Jezkova et al. 2016).

### Demographic Model Testing

To examine and compare the different models of the divergence of *P. cornutum* (riverine barrier, paleoclimate change, environmental gradients), we used MOMENTS (Jouganous et al. 2017) to simulate the 3D joint site frequency spectrum (JSFS) of genetic variation between the three populations based on results from our phylogenetic and population structure analyses. However, MOMENTS is based on the approximation of the discrete Wright–Fisher model, meaning that it is not appropriate to pool populations that may be genetically distinct (e.g., Plains cluster). Therefore, we used the same individuals in MOMENTS as in BPP analysis. For each data set (with and without KK104), we tested ten 3D models that were based on various aspects of divergence previously hypothesized for species in the region ranging from simple models with no gene flow to more complex models involving multiple time periods and varying degrees of gene flow between populations (fig. 6). We examined the possibility of river barriers preventing gene flow between adjacent populations, divergence in isolation with subsequent secondary contact, and various combinations involving models with allopatric and subsequent parapatric divergence along ecological clines (Schield et al. 2015; Jezkova et al. 2016; Myers et al. 2019).

The program easySFS (https://github.com/isaacovercast/easySFS, last accessed December 1, 2021) was used to determine the dimensions that would maximize segregating sites shared between samples when creating the folded JSFS; we also retained one SNP per locus to minimize linkage disequilibrium. MOMENTS is an efficient method of simulating the evolution of an allele frequency spectrum over time using differential equations. The basis of MOMENTS is similar to the diffusion approximation approach utilized in the program ∂a∂i and many of the models we tested were adapted from previously developed ∂a∂i and MOMENTS models (Gutenkunst et al. 2009; Portik et al. 2017; Leaché et al. 2019). For all models, we performed consecutive rounds of optimization with multiple replicates using the best scoring parameter (highest log-likelihood) estimates to base searches in the subsequent round (Portik et al. 2017; Leaché et al. 2019). Default settings in moments_pipeline (https://github.com/dportik/moments_pipeline, last accessed December 1, 2021) were used (replicates=10, 20, 30, 40; maxiter=3, 5, 10, 15; fold=3, 2, 2, 1), and we optimized parameters using optimize_log_fmin, a simplex (a.k.a. amoeba) method in terms of log parameters. Optimized parameter sets of each replicate were used to simulate the 3D-JSFS, and the multinomial approach was used to estimate the log-likelihood of the 3D-JSFS given the model. We ranked models according to AIC (lowest to highest) and estimated the standard deviation for each parameter using the Godambe Information Matrix with bootstrapped spectra. Finally, we determined the best model by comparing the two top ranked models for each data set using a likelihood-ratio test if they were nested. It should be noted that although that practices that we employed are common (e.g., selecting one SNP per locus, projecting down the JSFS), they can influence demographic inference. Projecting down the JSFS can result in composite likelihoods which can cause statistics such as AIC and BIC to favor more complex models (Gao and Song 2010; Coffman et al. 2016).

### Gene Flow and the Multispecies Coalescent with Introgression Model

Because several of our analyses suggested that gene flow was important throughout the evolutionary history of *P. cornutum*, we utilized the MSci in BPP (Flouri et al. 2020) to estimate introgression probabilities and reassess how divergence times and population sizes are affected when gene flow is explicitly modeled. Parameters and prior settings were virtually identical to the previous BPP analyses with a few exceptions. First, we used the best model from MOMENTS to specify a phylogenetic network (i.e., species tree with introgression events) for BPP to estimate parameters (i.e., *θ, τ*, and *φ*). This model included multiple reticulations in the species tree. For the introgression probability parameter (*φ*), we specified a beta prior of (1,1). We ran four independent analyses using a burnin of 200,000, followed by 500,000 samples that were taken every two generations. All BPP MSci analyses were run under a strict clock model (default) using BPP v. 4.3.0. Convergence was assessed by examining the trace plots in Tracer and checking for consistency between runs. All MSci analyses used the same 500 loci as the BPP MSC analyses. We performed analyses both with and without the admixed/outlier sample KK104. When included, KK104 was assigned to the Desert (DST) population following the results from the phylogenetic analyses.

### Species Distribution Modeling

We reconstructed the suitable climatic niche of *P. cornutum* for current climatic conditions and those of the LGM across the range of the species using ecological niche modeling. This methodology uses environmental data associated with occurrence records to estimate habitat suitability across the landscape by means of various program-specific algorithms (Elith et al. 2006). For occurrence data, we used our sampling localities, supplemented by occurrence records from the Vertnet (vertnet.org; queried May 1, 2018) and iNaturalist (iNaturalist.org; queried September 5, 2021) databases. All records with the coordinate uncertainty of 5 km and temperature outliers were removed, as well as all localities outside the known native range of the species and nonresearch grade records. This yielded 1,096 occurrence records. We then filtered the occurrence records using the R package spThin (Aiello-Lammens et al. 2015) to only include one occurrence record per 120 km. This filtering alleviated potential bias caused by unequal sampling effort (Merow et al. 2013) and differential coordinate access restrictions between states. This yielded 169 occurrence records used to inform the models.

We derived the current climatic niche of the species using 19 bioclimatic variables with resolution of 30 s (∼1 km) from the WorldClim data set (Hijmans et al. 2005). We derived the LGM climatic niche for *P. cornutum* using two simulation models of the LGM climate: community climate system model (CCSM ver. 3; Otto-Bliesner et al. 2006) with a resolution of 1°, and the model for interdisciplinary research on climate (MIROC ver. 3.2; Sugiyama et al. 2010) with an original spatial resolution of 1.4°×0.5° (Braconnot et al. 2007). These original climatic variables have been downscaled to the spatial resolution of 2.5 min (under the assumption of high spatial autocorrelation) and converted to bioclimatic variables (Hijmans et al. 2005; Peterson and Nyári 2008). These two models both indicate colder and wetter climate during the LGM. However, the CCSM model predicts lower values across temperature variables whereas the MIROC model predicts higher values across precipitation variables (see Jezkova et al. [2016]). We constructed climatic niche models for each climatic data set in the program MAXENT v. 3.3.3k (Phillips et al. 2006) using the R packages ENMeval (Muscarella et al. 2014) and dismo (Hijmans et al. 2015). MAXENT estimates relative probabilities of the presence of species within defined geographic spaces, with high probabilities indicating suitable environmental conditions (Phillips et al. 2006; Phillips and Dudík 2008). We used 1,000 background points randomly extracted from a polygon drawn around the occurrence records and expanded by two degrees in all directions. This selection of background points was chosen to exclude distant areas with very different environmental conditions, following recommendations by Merow et al. (2013). We explored values for the regularization multiplier (rm) between 0.5 and 4 (by increments of 0.5) and all combinations of available features (i.e., linear, quadratic, product, threshold, and hinge). We ran 3-fold cross-validation replicates to choose a model with the best fit, as assessed by the lowest AICc value. The best-fitting model for each climatic data set was visualized using logistic probability values (Merow et al. 2013). PCA analyses were also performed for current climate niche space occupied by the three and five genetic clusters derived from sNMF population structure and phylogenetic analyses and utilizing the 19 bioclimatic variables. 

## Supplementary Material

Supplementary data are available at *Genome Biology and Evolution* online.

## Supplementary Material

evab260_Supplementary_Data
