# Correction to: Genome-Scale Data Reveal Deep Lineage Divergence and a Complex Demographic History in the Texas Horned Lizard (*Phrynosoma cornutum*) throughout the Southwestern and Central United States

**DOI:** 10.1093/gbe/evac117

**Published:** 2022-08-18

This is a correction to: Nicholas Finger, Keaka Farleigh, Jason T Bracken, Adam D. Leaché, Olivier François, Ziheng Yang, Tomas Flouri, Tristan Charran, Tereza Jezkova, Dean A. Williams, Christopher Blair, Genome-Scale Data Reveal Deep Lineage Divergence and a Complex Demographic History in the Texas Horned Lizard (*Phrynosoma cornutum*) throughout the Southwestern and Central United States, *Genome Biology and Evolution*, Volume 14, Issue 1, January 2022, evab260, https://doi.org/10.1093/gbe/evab260.

In the originally published version of this manuscript, the individual data points were missing from Figure S5 in Supplementary Material.

This error has been corrected online.

