# Supplementary material for: Genome-Scale Data Reveal Deep Lineage Divergence and a Complex Demographic History in the Texas Horned Lizard (Phrynosoma cornutum) throughout the Southwestern and Central United States
Source: Genome Biol Evol. 2021 Nov 26;14(1):evab260. doi: 10.1093/gbe/evab260 (PMC8735750; doi:10.1093/gbe/evab260)
Supplement: evab260_Supplementary_Data [file evab260_supplementary_data.docx]

**Supplementary Material**

**Genome-scale data reveal deep lineage divergence and a complex demographic history in the Texas horned lizard (*Phrynosoma cornutum*) throughout the southwestern and central US**

Nicholas Finger, Keaka Farleigh, Jason Bracken, Adam D. Leaché, Olivier François, Ziheng Yang, Tomas Flouri, Tristan Charran, Tereza Jezkova, Dean A. Williams, Christopher Blair

**Supplementary Tables**

**Table S1.** Sampling data for all Texas horned lizards (*Phrynosoma cornutum*) and the outgroup (*P. solare*) used in this study. Raw total reads indicates the number of raw nGBS reads (total forward and reverse) for each sample. Loci in assembly shows the number of loci recovered from each sample after processing in ipyrad. Samples FHSM16593 and FHSM16898 were excluded from subsequent analyses due to a low number of reads. NA = Not Applicable.

| Species | ID | Plate_ID | Assembly_ID | Country | State | County | Lat | Long | Raw total reads | Loci in assembly |
| --- | --- | --- | --- | --- | --- | --- | --- | --- | --- | --- |
| *Phrynosoma cornutum* | CAS223566 | A1 | CAS223566 | USA | Colorado | Otero Co. | 37.70319 | -103.41536 | 2,628,492 | 19,250 |
| *Phrynosoma cornutum* | YPM18939 | A2 | YPM18939 | USA | New Mexico | Hidalgo County | 31.943009 | -108.869931 | 3,139,608 | 27,496 |
| *Phrynosoma cornutum* | MVZ238582 | A3 | MVZ238582 | USA | Arizona | Cochise County | 31.91259 | -109.15076 | 2,557,172 | 17,713 |
| *Phrynosoma cornutum* | FHSM9065 | A4 | FHSM9065 | USA | Kansas | Ellis | 39.1137 | -99.42567 | 4,042,644 | 36,182 |
| *Phrynosoma cornutum* | FHSM8954 | A5 | FHSM8954 | USA | Kansas | Russell | 39.0307 | -99.00036 | 4,118,078 | 35,328 |
| *Phrynosoma cornutum* | FHSM16593 | A6 | FHSM16593 | USA | New Mexico | Hidalgo | 32.61504 | -108.98934 | 171,164 | NA |
| *Phrynosoma cornutum* | CAS228870 | B1 | CAS228870 | USA | New Mexico | Luna Co. | 32.3675 | -107.8428333 | 3,496,728 | 31,894 |
| *Phrynosoma cornutum* | YPM18934 | B2 | YPM18934 | USA | New Mexico | Hidalgo County | 31.94322 | -108.88875 | 3,113,348 | 28,861 |
| *Phrynosoma cornutum* | MVZ269788 | B3 | MVZ269788 | USA | Oklahoma | Harper County | 36.9586 | -99.9101 | 2,552,102 | 22,035 |
| *Phrynosoma cornutum* | FHSM8541 | B4 | FHSM8541 | USA | Kansas | Harper | 37.02347 | -97.83816 | 3,586,546 | 34,503 |
| *Phrynosoma cornutum* | FHSM11180 | B5 | FHSM11180 | USA | Kansas | Seward | 37.13912 | -100.78118 | 4,396,558 | 38,822 |
| *Phrynosoma cornutum* | FHSM16233 | B6 | FHSM16233 | USA | Oklahoma | Beckham | 35.15165421 | -99.85324662 | 4,135,670 | 38,433 |
| *Phrynosoma cornutum* | CAS228873 | C1 | CAS228873 | USA | Arizona | Cochise Co. | 31.62443056 | -109.2074889 | 3,026,500 | 25,894 |
| *Phrynosoma cornutum* | YPM18947 | C2 | YPM18947 | USA | Arizona | Cochise County | 31.90903 | -109.11222 | 3,097,846 | 29,694 |
| *Phrynosoma cornutum* | FHSM9128 | C3 | FHSM9128 | USA | Kansas | Meade | 37.21475 | -100.46707 | 4,976,888 | 42,652 |
| *Phrynosoma cornutum* | FHSM8606 | C4 | FHSM8606 | USA | Kansas | Kiowa | 37.4661 | -99.10786 | 4,211,224 | 36,869 |
| *Phrynosoma cornutum* | FHSM11375 | C5 | FHSM11375 | USA | Kansas | Seward | 37.12771 | -100.7822 | 3,992,240 | 37,881 |
| *Phrynosoma cornutum* | FHSM16007 | C6 | FHSM16007 | USA | Oklahoma | Cimarron | 36.81620278 | -102.8517467 | 3,662,888 | 35,591 |
| *Phrynosoma cornutum* | CAS228872 | D1 | CAS228872 | USA | Arizona | Cochise Co. | 31.65231944 | -109.1589694 | 3,050,294 | 27,856 |
| *Phrynosoma cornutum* | OCGR14021 | D2 | OCGR14021 | USA | Oklahoma | Canadian | 38.696014 | -98.0207 | 3,532,950 | 31,258 |
| *Phrynosoma cornutum* | FHSM8542 | D3 | FHSM8542 | USA | Kansas | Barber | 37.13965 | -98.69531 | 3,786,694 | 36,435 |
| *Phrynosoma cornutum* | FHSM8607 | D4 | FHSM8607 | USA | Kansas | Kiowa | 37.51368 | -99.15667 | 4,440,656 | 39,848 |
| *Phrynosoma cornutum* | FHSM9010 | D5 | FHSM9010 | USA | Kansas | Stanton | 37.51959 | -102.01588 | 4,660,100 | 40,689 |
| *Phrynosoma cornutum* | FHSM16232 | D6 | FHSM16232 | USA | Oklahoma | Greer | 35.0942235 | -99.78271819 | 3,620,344 | 35,256 |
| *Phrynosoma cornutum* | CAS223567 | E1 | CAS223567 | USA | Colorado | Las Animas Co. | 37.589185 | -103.329031 | 2,790,664 | 24,773 |
| *Phrynosoma cornutum* | OCGR9297 | E2 | OCGR9297 | USA | Oklahoma | Cimarron | 36.6629 | -102.78673 | 2,127,180 | 12,266 |
| *Phrynosoma cornutum* | FHSM11825 | E3 | FHSM11825 | USA | Kansas | Barber | 37.09546 | -98.96539 | 3,577,724 | 30,615 |
| *Phrynosoma cornutum* | FHSM9111 | E4 | FHSM9111 | USA | Kansas | Lincoln | 39.0794 | -98.1511 | 3,620,426 | 33,438 |
| *Phrynosoma cornutum* | FHSM16492 | E5 | FHSM16492 | USA | Kansas | Stanton | 37.486559 | -102.041782 | 1,129,208 | 4,757 |
| *Phrynosoma cornutum* | FHSM16223 | E6 | FHSM16223 | USA | Oklahoma | Harmon | 34.95179125 | -99.9145693 | 3,717,844 | 35,981 |
| *Phrynosoma cornutum* | CAS223561 | F1 | CAS223561 | USA | Colorado | Otero Co. | 37.76283333 | -103.5785 | 3,254,194 | 31,438 |
| *Phrynosoma cornutum* | OCGR6643 | F2 | OCGR6643 | USA | Oklahoma | Ellis | 35.8934 | -99.6637 | 2,534,122 | 21,926 |
| *Phrynosoma cornutum* | FHSM16898 | F3 | FHSM16898 | USA | Kansas | Chautauqua | 37.09865 | -96.26519 | 482,518 | NA |
| *Phrynosoma cornutum* | FHSM8605 | F4 | FHSM8605 | USA | Kansas | Meade | 37.0531 | -100.10749 | 3,626,724 | 34,054 |
| *Phrynosoma cornutum* | FHSM11371 | F5 | FHSM11371 | USA | Kansas | Stevens | 37.07085 | -101.4834 | 3,299,466 | 28,957 |
| *Phrynosoma cornutum* | FHSM16024 | F6 | FHSM16024 | USA | Oklahoma | Harper | 36.64099493 | -99.4764639 | 4,042,414 | 36,568 |
| *Phrynosoma cornutum* | YPM18957 | G1 | YPM18957 | USA | New Mexico | Hidalgo County | 31.932925 | -108.969717 | 3,248,856 | 30,276 |
| *Phrynosoma cornutum* | MVZ161507 | G2 | MVZ161507 | USA | New Mexico | Dona Ana County | 32.312222 | -106.777779 | 3,031,026 | 26,703 |
| *Phrynosoma cornutum* | FHSM8581 | G3 | FHSM8581 | USA | Kansas | Clark | 37.19376 | -99.87469 | 3,273,952 | 31,369 |
| *Phrynosoma cornutum* | FHSM11559 | G4 | FHSM11559 | USA | Kansas | Osborne | 39.17528 | -98.8996 | 3,779,880 | 36,322 |
| *Phrynosoma cornutum* | FHSM16889 | G5 | FHSM16889 | USA | Kansas | Stevens | 37.054774 | -101.483188 | 3,696,916 | 34,764 |
| *Phrynosoma cornutum* | FHSM17437 | G6 | FHSM17437 | USA | Kansas | Elk | 37.567365 | -96.495885 | 2,717,418 | 24,535 |
| *Phrynosoma cornutum* | YPM18933 | H1 | YPM18933 | USA | Arizona | Cochise County | 31.90903 | -109.11222 | 3,058,390 | 27,171 |
| *Phrynosoma cornutum* | MVZ252854 | H2 | MVZ252854 | USA | New Mexico | Dona Ana County | 32.53023333 | -106.7740167 | 2,624,244 | 20,976 |
| *Phrynosoma cornutum* | FHSM9126 | H3 | FHSM9126 | USA | Kansas | Comanche | 37.25453 | -99.11038 | 4,300,416 | 37,922 |
| *Phrynosoma cornutum* | FHSM8038 | H4 | FHSM8038 | USA | Kansas | Russell | 38.7994 | -99.0196 | 3,826,740 | 34,920 |
| *Phrynosoma cornutum* | FHSM16814 | H5 | FHSM16814 | USA | New Mexico | Grant | 32.558056 | -107.984478 | 3,275,088 | 29,636 |
| *Phrynosoma cornutum* | 10R2L8L | P1-10R2L8L | 10R2L8L | USA | Texas | Dimmit/La Salle | 28.32603 | -99.407555 | 3,633,126 | 20,955 |
| *Phrynosoma cornutum* | 5R7R6L | P1-5R7R6L | 5R7R6L | USA | Texas | Potter | 35.419743 | -101.947293 | 4,030,164 | 23,331 |
| *Phrynosoma cornutum* | 7R10L | P1-7R10L | 7R10L | USA | Texas | Dimmit/La Salle | 28.32603 | -99.407555 | 2,858,336 | 18,544 |
| *Phrynosoma cornutum* | ABGF01 | P1-AGBF01 | ABGF01 | USA | Texas | Brisco | 34.547778 | -100.950556 | 9,410,592 | 33,297 |
| *Phrynosoma cornutum* | ABGF11 | P1-AGBF11 | ABGF11 | USA | Texas | Motley | 34.1525 | -100.704444 | 4,842,856 | 23,150 |
| *Phrynosoma cornutum* | BL2 | P1-BL2 | BL2 | USA | Texas | Midland | 31.912383 | -102.069583 | 2,614,366 | 16,903 |
| *Phrynosoma cornutum* | BS2 | P1-BS2 | BS2 | USA | Texas | Nolan | 32.15489916 | -100.4307774 | 12,423,608 | 30,309 |
| *Phrynosoma cornutum* | CO14 | P1-CO14 | CO14 | USA | Colorado | Prowers | 37.659408 | -102.544595 | 4,349,506 | 28,893 |
| *Phrynosoma cornutum* | CO2 | P1-CO2 | CO2 | USA | Colorado | Baca | 37.235833 | -102.724035 | 5,879,784 | 35,553 |
| *Phrynosoma cornutum* | K10 | P1-K10 | K10 | USA | Texas | Karnes | 28.888123 | -97.898467 | 5,758,698 | 24,942 |
| *Phrynosoma cornutum* | K24 | P1-K24 | K24 | USA | Texas | Karnes | 28.890415 | -97.897358 | 5,409,790 | 24,584 |
| *Phrynosoma cornutum* | K71 | P1-K71 | K71 | USA | Texas | Karnes | 28.810068 | -97.845914 | 5,032,512 | 26,854 |
| *Phrynosoma cornutum* | KK104 | P1-KK104 | KK104 | USA | Texas | Brewster | 30.0032 | -103.488 | 4,900,104 | 23,031 |
| *Phrynosoma cornutum* | KL05 | P1-KL05 | KL05 | USA | Texas | Midland | 31.893113 | -102.327828 | 4,283,458 | 23,307 |
| *Phrynosoma cornutum* | MA123 | P1-MA123 | MA123 | USA | Texas | Cottle | 34.117351 | -100.344618 | 8,468,242 | 29,485 |
| *Phrynosoma cornutum* | MA133 | P1-MA133 | MA133 | USA | Texas | Cottle | 34.117351 | -100.344618 | 2,460,288 | 14,402 |
| *Phrynosoma cornutum* | MA166 | P1-MA166 | MA166 | USA | Texas | Cottle | 34.117351 | -100.344618 | 4,695,164 | 24,657 |
| *Phrynosoma cornutum* | MA182 | P1-MA182 | MA182 | USA | Texas | Cottle | 34.117351 | -100.344618 | 10,111,700 | 30,331 |
| *Phrynosoma cornutum* | PhCo1 | P1-Pc1 | Pc1 | USA | New Mexico | Tucumcari | 35.1833 | -103.663 | 5,216,602 | 25,629 |
| *Phrynosoma cornutum* | PhCo2 | P1-Pc2 | Pc2 | USA | New Mexico | Tucumcari | 35.1833 | -103.663 | 6,503,754 | 25,430 |
| *Phrynosoma cornutum* | PhCo3 | P1-Pc3 | Pc3 | USA | New Mexico | Tucumcari | 35.1833 | -103.663 | 7,872,410 | 28,340 |
| *Phrynosoma cornutum* | PhCo4 | P1-Pc4 | Pc4 | USA | New Mexico | Tucumcari | 35.1833 | -103.663 | 7,185,118 | 27,746 |
| *Phrynosoma cornutum* | PhCo5 | P1-Pc5 | Pc5 | USA | New Mexico | Carlsbad | 32.28549 | -104.63 | 3,531,324 | 20,257 |
| *Phrynosoma cornutum* | PhCo6 | P1-Pc6 | Pc6 | USA | New Mexico | Carlsbad | 32.28812 | -104.608 | 4,705,198 | 23,426 |
| *Phrynosoma cornutum* | UTA R-65008 | P1-UTA65008 | UTA65008 | USA | Texas | Briscoe | 34.4 | -101.05 | 5,747,492 | 26,158 |
| *Phrynosoma cornutum* | MA227 | P1-MA227 | MA227 | USA | Texas | Cottle | 34.117351 | -100.344618 | 11,323,318 | 34,095 |
| *Phrynosoma cornutum* | MA230 | P1-MA230 | MA230 | USA | Texas | Cottle | 34.117351 | -100.344618 | 5,139,164 | 25,251 |
| *Phrynosoma cornutum* | MA69 | P1-MA69 | MA69 | USA | Texas | Cottle | 34.117351 | -100.344618 | 8,680,360 | 27,959 |
|  |  |  |  |  |  |  |  |  |  |  |
| *Phrynosoma solare* | FHSM9405 | H6 | FHSM9405 | USA | Arizona | Pima | 32.32778 | -111.2175 | 4,069,032 | 15,278 |

**Table S2**: Measures of genetic variation among populations of *Phrynosoma cornutum* based on SNP data. Between population values are presented on the upper diagonal in Nei’s genetic distances. The lower diagonal values represent pairwise population differentiation (F_st_), 95% confidence intervals are indicated in parentheses. Within population variation is presented in Nei’s genetic distances.

| Between-Populations | | | |  | Within-Populations | |
| --- | --- | --- | --- | --- | --- | --- |
|  | **Southern** | **Plains** | **Desert** |  | **Southern** | 0.113 |
| Southern | - | 0.047 | 0.191 |  | **Plains** | 0.094 |
| Plains | 0.096 (0.094-0.097) | - | 0.195 |  | **Desert** | 0.052 |
| Desert | 0.402 (0.399-0.405) | 0.494 (0.491-0.496) | - |  |  |  |

**Table S3**. Estimates of effective population sizes from multispecies coalescent (MSC) analyses in BPP. The 'Estimate' and 'Calibrated Estimate' columns indicate mean values obtained by combining two independent runs per data set. All raw parameter values are *1000. Absolute effective population sizes (*N*_e_) were obtained by assuming a per year mutation rate of 8 * 10^-10^ [(Perry et al., 2018)](https://www.zotero.org/google-docs/?IzXA1N) and a generation time of two years [(Jezkova et al., 2016)](https://www.zotero.org/google-docs/?WpWb9H). OG = outgroup; IG = ingroup; DST = Desert (pop 1); STH = Southern (pop 2); PLN = Plains (pop 3). non-admixed = runs excluding sample KK104 (from DST); admixed = runs including this individual. Estimates in bold italics indicate substantial differences between data sets. * = two peaks in the posterior.

| Run | Parameter | Estimate | 95% HPD lower | 95% HPD upper |  | Calibrated Estimate | 95% HPD lower | 95% HPD upper |
| --- | --- | --- | --- | --- | --- | --- | --- | --- |
| non-admixed | *θ*_OG_ | 1.301 | 0.596 | 2.128 |  | 203,281 | 93,125 | 332,500 |
| ***non-admixed*** | ***θ_DST_*** | ***1.495*** | ***1.279*** | ***1.701*** |  | ***233,593*** | ***199,843*** | ***265,781*** |
| non-admixed | *θ_STH_* | 3.686 | 2.186 | 5.231 |  | 575,937 | 341,562 | 817,343 |
| non-admixed | *θ_PLN_* | 1.007 | 0.618 | 1.42 |  | 157,343 | 96,562 | 221,875 |
| ***non-admixed*** | ***θ_Root_*** | ***23.55*** | ***15*** | ***33*** |  | ***3,679,687*** | ***2,343,750*** | ***5,156,250*** |
| ***non-admixed*** | ***θ_IG_*** | ***4.005*** | ***3.428*** | ***4.586*** |  | ***625,781*** | ***535,625*** | ***716,562*** |
| non-admixed | *θ*_STH+PLN_ | 5.956 | 4.86 | 7.064 |  | 930,625 | 759,375 | 1,103,750 |
|  |  |  |  |  |  |  |  |  |
| admixed | *θ*_OG_ | 1.231 | 0.511 | 2.112 |  | 192,343 | 79,843 | 330,000 |
| ***admixed*** | ***θ_DST_*** | ***2.228*** | ***1.936*** | ***2.532*** |  | ***348,125*** | ***302,500*** | ***395,625*** |
| admixed | *θ_STH_* | 3.521 | 2.093 | 5.005 |  | 550,156 | 327,031 | 782,031 |
| admixed | *θ_PLN_* | 1.036 | 0.621 | 1.467 |  | 161,875 | 97,031 | 229,218 |
| ****non-admixed*** | ***θ_Root_*** | ***16.46*** | ***0.499*** | ***26*** |  | ***2,571,875*** | ***77,968*** | ***4,062,500*** |
| ***non-admixed*** | ***θ_IG_*** | ***4.947*** | ***4.393*** | ***5.489*** |  | ***772,968*** | ***686,406*** | ***857,656*** |
| non-admixed | *θ*_STH+PLN_ | 6.051 | 4.672 | 7.474 |  | 945,468 | 730,000 | 1,167,812 |

**Table S4**. Parameter estimates from the lowest AIC run for each demographic model considered (see Fig. 6) for each data set (upper panel: non-admixed; lower panel: admixed) in the program MOMENTS; standard deviations for each parameter are in parentheses. Models with the lowest AIC are considered the best supported. Parameters are represented by population size (nu; *N_ref_* = theta/4µ where µ is mutation rate), migration rate (m; *M_ij_* = 2*N_ref_m_ij_*), and divergence time (T; T = 2*N_ref_*); the parameters presented here are unscaled. For example, nu1 represents the population size of populations 1 (DST), m12 represents the migration from population 1 into population 2 (e.g., DST into STH), and T1 represents the time interval from divergence to time T2; see Fig. 5 for a visualization. For models with symmetric migration, we report the parameter estimate twice (see split_sym_mig_all).

| non-admixed  data set |  |  |  |  |  |  |  |  |  |  |  |  |  |  |  |  |  |  |
| --- | --- | --- | --- | --- | --- | --- | --- | --- | --- | --- | --- | --- | --- | --- | --- | --- | --- | --- |
| Model | **log.likelihood** | **AIC** | **chi.squared** | **theta** | **nu1** | **nuA** | **nu2** | **nu3** | **m12** | **m13** | **m21** | **m23** | **m32** | **T1** | **T1a** | **T1b** | **T2** | **T3** |
| refugia_adj_2 | -249.14 | 514.28 | 226.15 | 36.63 | 1.86  (3.9e-3) | 2.60  (8e-4) | 2.59  (4.7e-3) | 2.01  (3.9e-3) | 0.03  (1e-4) |  | 0.03  (1e-4) | 0.37  (7e-4) | 0.37  (7e-4) | 5.02  (1.1e-2) |  |  | 0.31  (1e-4) |  |
| refugia_asymmig_adjacent | -248.95 | 517.90 | 198.72 | 113.36 | 0.56  (3e-4) | 0.83  (5e-4) | 1.62  (4.1e-3) | 0.81  (2e-4) | 0.22  (1e-4) |  | 0.05(3e-4) | 0.31  (2.7e-3) | 0.03  (2e-4) | 0.91  (8e-4) |  |  | 0.24  (5e-4) |  |
| refugia_barrier | -252.24 | 518.48 | 247.08 | 103.30 | 0.55  (1e-4) | 1.58  (1.1e-3) | 1.03  (2e-4) | 0.95  (3e-4) |  |  |  | 0.47  (3e-4) | 0.47  (3e-4) | 0.54  (4e-4) |  |  | 0.49  (1e-4) |  |
| split_nomig | -253.69 | 519.38 | 174.65 | 160.43 | 0.41  (2e-4) | 0.57  (4e-4) | 0.76  (1e-4) | 0.46  (1e-4) |  |  |  |  |  | 0.43  (3e-4) |  |  | 0.16  (1e-4) |  |
| refugia_adj_1 | -255.77 | 529.54 | 270.55 | 84.00 | 0.70  (4e-4) | 20.48  (5.1e-2) | 1.50  (1e-3) | 0.68  (3e-4) | 0.03  (1e-4) |  | 0.03  (1e-4) | 0.92  (3e-4) | 0.92  (3e-4) | 0.28  (1.5e-3) |  |  | 0.22  (1e-4) | 1.25  (6e-4) |
| refugia_adj_3 | -266.52 | 553.04 | 332.80 | 140.36 | 0.29  (1e-4) | 0.34  (2e-4) | 0.93  (6e-4) | 0.54  (2e-4) | 0.08  (4e-4) |  | 0.08  (4e-4) | 1.13  (5e-4) | 1.13  (5e-4) |  | 0.91  (5e-4) | 4.03  (4e-4) | 0.94  (5e-4) |  |
| split_asymmig_adjacent | -267.63 | 559.26 | 231.18 | 75.11 | 0.63  (6e-4) | 2.84  (5e-4) | 2.01  (7.2e-3) | 0.50  (7e-4) | 0.07  (2e-4) |  | 0.03(3e-4) | 0.42  (6.1e-3) | 2.63  (4.6e-3) | 0.05  (1e-4) |  |  | 2.32  (7.5e-3) |  |
| split_sym_mig_all | -290.35 | 600.70 | 264.19 | 171.67 | 0.30  (2.2e-3) | 0.12  (1.3e-4) | 2.52  (5e-4) | 0.26  (3e-4) | 0.04  (2.4e-3) | 0.28  (1e-4) | 0.04  (2.4e-3) | 2.54  (1.2e-2) | 2.54  (1.2e-2) | 0.22  (1.7e-2) |  |  | 0.49  (7.3e-3) |  |
| split_symmig_adjacent | -303.33 | 624.66 | 246.37 | 190.95 | 0.23  (1e-4) | 0.45  (1e-4) | 0.36  (1e-4) | 0.28  (1e-4) | 0.18  (2e-4) |  | 0.18  (2e-4) | 4.84  (3.2e-3) | 4.84  (3.2e-3) | 0.14  (1.4e-3) |  |  | 0.45  (1.7e-3) |  |
| refugia_symmig_all | -305.99 | 629.98 | 330.80 | 56.04 | 0.07  (2e-4) | 5.28  (1e-4) | 1.18  (1e-4) | 0.03  (3e-4) | 0.94  (2e-4) | 0.04  (1e-4) | 0.94  (2e-4) | 1.22  (5e-4) | 1.22  (5e-4) | 0.56  (6e-4) |  |  | 3.15  (1.1e-3) |  |
| admixed  data set |  |  |  |  |  |  |  |  |  |  |  |  |  |  |  |  |  |  |
| Model | **log.likelihood** | **AIC** | **chi.squared** | **theta** | **nu1** | **nuA** | **nu2** | **nu3** | **m12** | **m13** | **m21** | **m23** | **m32** | **T1** | **T1a** | **T1b** | **T2** | **T3** |
| refugia_barrier | -362.19 | 738.38 | 277.12 | 324.17 | 0.54  (3e-4) | 0.38  (1.6e-3) | 1.57  (8e-4) | 0.62  (2e-4) |  |  |  | 1.20  (8e-4) | 1.20  (8e-4) | 0.49  (2e-4) |  |  | 0.20  (7e-4) |  |
| refugia_adj_2 | -367.39 | 750.78 | 257.94 | 236.86 | 0.77  (1.9e-3) | 1.18  (5.3e-3) | 1.57  (2.3e-3) | 1.09  (1.9e-3) | 0.15  (1e-4) |  | 0.15  (1e-4) | 0.31  (2.4e-3) | 0.31  (2.4e-3) | 1.08  (5.1e-3) |  |  | 0.38  (4e-4) |  |
| refugia_adj_1 | -380.41 | 778.82 | 371.24 | 160.46 | 1.19  (6e-3) | 1.34  (2e-2) | 2.13  (9.4e-3) | 1.74  (1e-2) | 0.05  (1e-4) |  | 0.05  (1e-4) | 0.40  (2.1e-3) | 0.40  (2.1e-3) | 0.84  (1.3e-2) |  |  | 0.61  (1.3e-3) | 0.65  (5e-4) |
| refugia_adj_3 | -379.66 | 779.32 | 301.82 | 139.00 | 1.54  (1.9e-3) | 1.15  (1.1e-3) | 2.43  (2.4e-3) | 1.11  (9e-4) | 0.06  (1e-4) |  | 0.06  (1e-4) | 0.71  (6e-4) | 0.71  (6e-4) |  | 0.02  (1e-4) | 0.66  (3e-4) | 2.39  (2.6e-3) |  |
| split_asymmig_adjacent | -387.52 | 799.04 | 298.65 | 415.02 | 0.39  (1e-4) | 0.36  (8e-4) | 0.46  (1e-4) | 0.68  (9e-4) | 0.04  (2e-4) |  | 0.06  (2e-4) | 3.36  (1.3e-3) | 0.01(2e-4) | 1.74  (2.4e-3) |  |  | 0.53  (4e-4) |  |
| split_nomig | -414.18 | 840.36 | 428.95 | 310.29 | 0.66  (1.3e-3) | 0.94  (2.2e-3) | 13.1  (3.2e-3) | 0.49  (3e-4) |  |  |  |  |  | 0.66  (1.6e-3) |  |  | 0.66  (1.6e-3) |  |
| split_sym_mig_all | -425.44 | 870.88 | 323.88 | 1026.73 | 0.13  (1e-4) | 0.13  (1e-4) | 0.36  (1e-4) | 0.32  (1e-4) | 0.34  (2e-4) | 0.05  (3e-4) | 0.34  (2e-4) | 0.26  (1e-4) | 0.26  (1e-4) | 1.21  (5e-4) |  |  | 0.11  (2e-4) |  |
| refugia_asymmig_adjacent | -432.25 | 884.50 | 412.79 | 141.37 | 0.89  (1e-3) | 14.08  (1.9e-2) | 1.25  (1e-4) | 1.26  (3e-4) | 0.10  (3e-4) |  | 0.14  (2e-4) | 2.08  (1e-4) | 0.59(5e-4) | 2.14  (3e-4) |  |  | 0.90  (8.9e-3) |  |
| split_symmig_adjacent | -487.17 | 992.34 | 591.38 | 193.82 | 0.52  (6e-4) | 10.94  (7.1e-3) | 0.89(5e-4) | 0.82(7e-4) | 0.06(2e-4) |  | 0.06(2e-4) | 0.45(4e-4) | 0.45(4e-4) | 1.26(4.6e-3) |  |  | 0.32  (5e-4) |  |
| refugia_symmig_all | -604.40 | 1226.80 | 664.22 | 1335.13 | 0.07  (1e-4) | 5.28  (1.3e-1) | 1.18(1e-4) | 0.03(1e-4) | 0.94(1e-4) | 1.40(2e-4) | 0.94(1e-4) | 21.89(1e-4) | 21.89(1e-4) | 1.56(1.4e-2) |  |  | 13.02(2e-4) |  |

**Supplementary Figures**

**Fig. S1.** Majority-rule bootstrap consensus tree from coalescent analysis in SVDquartets. Tree was inferred using an unlinked SNP matrix (54,634 SNPs). Branch values represent nonparametric bootstrap support (100 replicates).

**
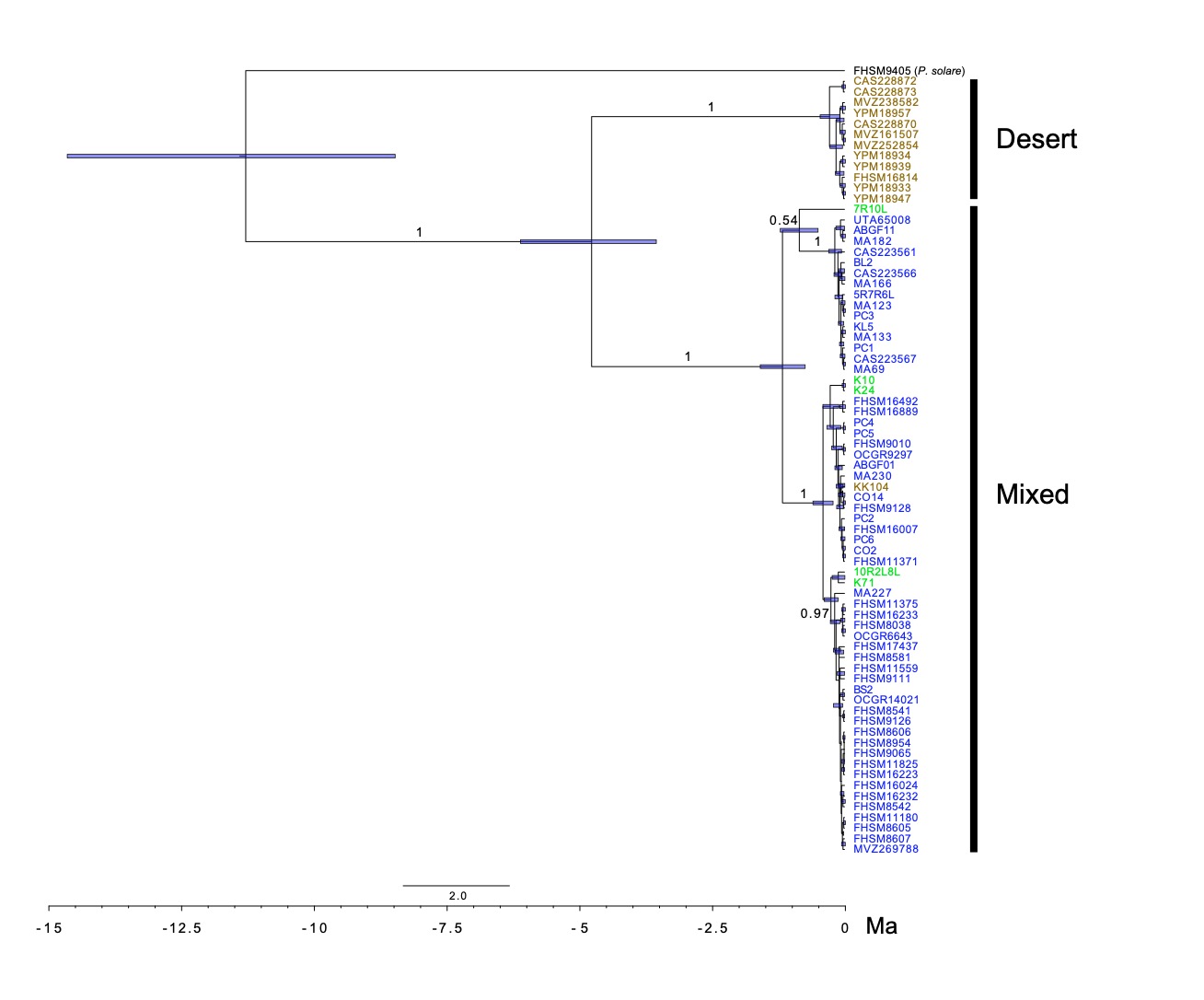
**

**Fig. S2.** Bayesian maximum clade credibility (MCC) tree from BEAST based on ND1, tRNA leucine, tRNA isoleucine, tRNA glutamine, and portions of 16S and tRNA methionine. Sample tips are color coded to match those in Fig. 2. Values at nodes represent posterior probability values. Node bars indicate 95% HPD for mean node heights. The horizontal scale bar represents time in millions of years (Ma).

**Fig. S3.** sNMF admixture results for varying values of *K*. Each bar represents a sampled individual, with colors indicating the proportion of ancestry assigned to a particular ancestral population. Note that the outgroup (*P. solare*) was removed prior to analysis.

**Fig. S4.** Geographic distribution of the five ancestral populations of *P. cornutum* as inferred by sNMF. Colors correspond to those in Figs. 2 and S3.

**
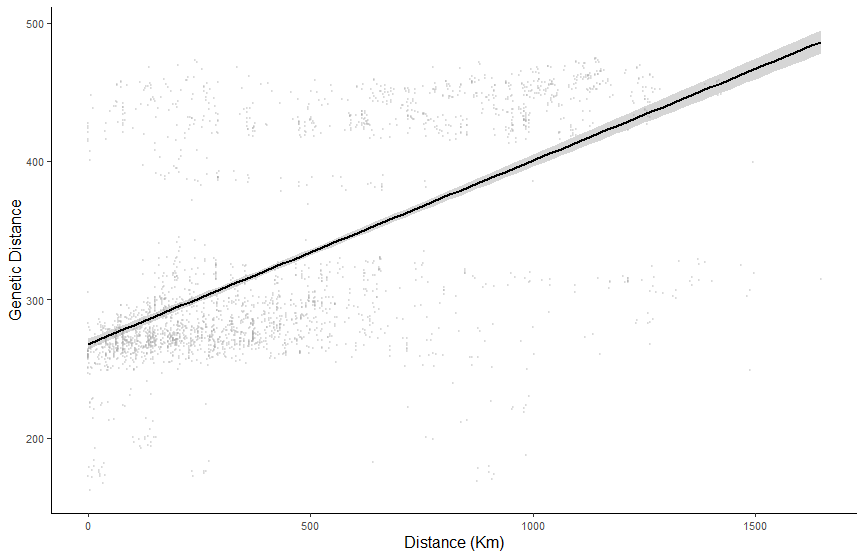
**

**Fig S5**. Relationship between genetic and geographic distances for all individuals (grey points). The black solid line is linear model fit, with the grey shaded regions representing the 95% confidence interval.

**Fig. S6.** Number of hits for different bioclimatic variables as inferred using LFMM. The largest number of SNPs were statistically correlated with bio9 (mean temperature of the driest quarter) and bio17 (precipitation of the driest quarter). bio1 = Annual Mean Temperature; bio2 = Mean Diurnal Range; bio3 = Isothermality; bio4 = Temperature Seasonality; bio5 = Max Temperature of Warmest Month; bio6 = Min Temperature of Coldest Month; bio7 = Temperature Annual Range; bio8 = Mean Temperature of Wettest Quarter; bio9 = Mean Temperature of Driest Quarter; bio10 = Mean Temperature of Warmest Quarter; bio11 = Mean Temperature of Coldest Quarter; bio12 = Annual Precipitation; bio13 = Precipitation of Wettest Month; bio14 = Precipitation of Driest Month; bio15 = Precipitation Seasonality; bio16 = Precipitation of Wettest Quarter; bio17 = Precipitation of Driest Quarter; bio18 = Precipitation of Warmest Quarter; bio19 = Precipitation of Coldest Quarter.

**
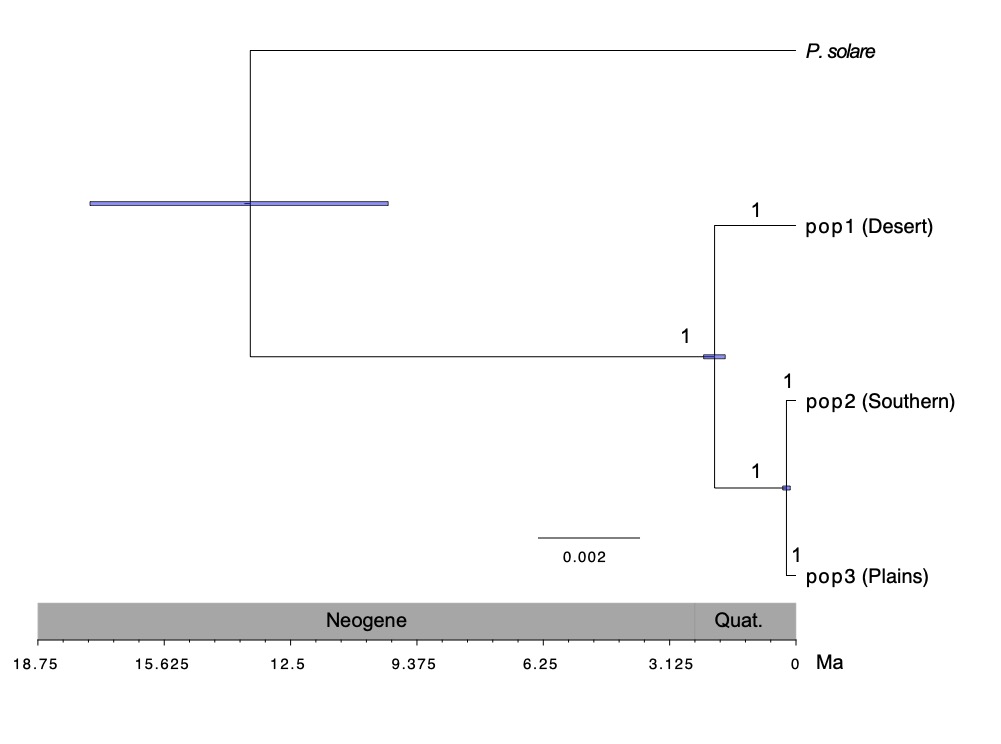
**

**Fig. S7**. Divergence time estimates of *Phrynosoma cornutum* populations based on MSC analysis of nGBS data in BPP (500 loci). Parameter estimates were based on data sets that excluded the highly admixed sample KK104. Values at tips represent posterior probability support for species/population. Values adjacent to nodes represent posterior probability support for phylogenetic relationships.

**Fig. S8**. Two local peaks in the posterior for parameters in the MSci model in the BPP analysis of the admixed data including KK104. The two peaks represent two hypotheses that have nearly equal support from the data, due to the species tree being nearly a trichotomy. Thickness of lines represent strength of gene flow/introgression. See Table 2 for the parameter estimates. See legend to Fig. 7 for additional information.

**Fig. S9.** Four unidentifiable models due to introgression events at nodes *A* and *B* and at *C* and *D*. Thickness of the horizontal (introgression) lines reflect estimates of introgression probabilities for Peak 1 using the non-admixed dataset from Table 2: *α* = *ϕ_C_* = 0.065, *β* = *ϕ_D_* = 0.935, *γ* = *ϕ_A_* = 0.868, and *δ* = *ϕ_B_* = 0.090. Tree *S*_1_ is the model of Fig. 7a, while trees *S*_2_, *S*_3_, and *S*_4_ are alternative models that are unidentifiable by genomic sequence data. The outgroup and node *R* of Fig. 7 are omitted for simplicity. The four models predict the same distribution of gene trees and coalescent times and are thus unidentifiable using genetic sequence data. One way of seeing this unidentifiability or equivalence of the four models is to confirm that the four models predict the same major genealogical routes when we trace the sampled sequences backwards in time and follow the major parental paths at each hybridization node (indicated by the introgression probabilities). Thus we identify the most recent common ancestor to be node *C* for populations 2 and 3 and node *B* for populations 1 and 2 (or 3).
